# Pesticide exposure and child growth in low- and middle-income countries: A systematic review

**DOI:** 10.1016/j.envres.2022.114230

**Published:** 2022-09-07

**Authors:** Lilia Bliznashka, Aditi Roy, Lindsay M. Jaacks

**Affiliations:** a Global Academy of Agriculture and Food Systems, University of Edinburgh, Alexander Robertson Building, Easter Bush Campus, Midlothian, EH25 9RG, UK; b Centre for Environmental Health, Public Health Foundation of India, Plot No. 47, Sector 44, Institutional Area Gurugram, 122002, India

**Keywords:** Organophosphates, Organochlorines, Pyrethroids, Birth weight, Prenatal exposure, Low birth weight

## Abstract

**Background:**

In low- and middle-income countries (LMICs), pesticides are widely used in agricultural and residential settings. Little is known about how pesticides affect child growth.

**Objectives:**

To systematically review and synthesise the evidence on the associations between pesticide exposure and adverse birth outcomes and/or impaired postnatal growth in children up to 5 years of age in LMICs.

**Methods:**

We searched 10 databases from inception through November 2021. We included cohort and cross-sectional studies investigating associations between self-reported or measured prenatal or postnatal pesticide exposure and child growth (postnatal child linear/ponderal growth, and/or birth outcomes). Two researchers screened studies, extracted data, and assessed certainty using GRADE. The protocol was preregistered with PROSPERO (CRD42021292919).

**Results:**

Of 939 records retrieved, 31 studies met inclusion criteria (11 cohort, 20 cross-sectional). All studies assessed prenatal exposure. Twenty-four studies reported on birth weight. Four found positive associations with organochlorines (0.01–0.25 standardised mean difference (SMD)) and two found negative associations (–0.009 SMD to –55 g). Negative associations with organophosphates (–170 g, n = 1) and pyrethroids (–97 to –233 g, n = 2) were also documented. Two (out of 15) studies reporting on birth length found positive associations with organochlorines (0.21–0.25 SMD) and one found negative associations (–0.25 to –0.32 SMD). Organophosphate exposure was negatively associated with birth length (–0.37 cm, n = 1). Organophosphate exposure was also associated with higher risk/prevalence of low birth weight (2 out of nine studies) and preterm birth (2 out of six studies). Certainty of the evidence was “very low” for all outcomes.

**Conclusion:**

The limited literature from LMICs shows inconclusive associations between prenatal pesticide exposure, child growth, and birth outcomes. Studies with accurate quantitative data on exposure to commonly used pesticides in LMICs using consistent methodologies in comparable populations are needed to better understand how pesticides influence child growth.

## 1 Introduction

Pesticides are widely used in agricultural and residential settings globally (FAO, 2021; World Health Organization and Food and Agriculture Organization of the United Nations, 2019). In some low- and middle-income countries (LMICs), like those in sub-Saharan Africa, South and Southeast Asia, pesticide use per cropland area remains lower than in high-income countries (FAO, 2021). However, pesticide exposure can be substantially higher in LMICs than high-income countries and populations may be exposed to more toxic chemicals in these settings (World Health Organization and Food and Agriculture Organization of the United Nations, 2019; Pan International, 2021). This stems from: (1) the fact that high-income countries continue to export pesticides that have been banned for use in their own countries; (2) fewer regulations in some LMICs (in place and/or properly enforced); and (3) unsafe pesticide handling, storage, application, and disposal practices which are more commonly used in many LMICs than in high-income countries (World Health Organization and Food and Agriculture Organization of the United Nations, 2019; Pan International, 2021). Dietary intake of pesticide residues from contaminated foods and pesticide use for water chlorination are also among the primary routes of exposure (Mostafalou and Abdollahi, 2017).

Children are especially vulnerable to the harmful effects of pesticides due to their increased exposure relative to their body weight, behavioural factors (they spend more time on the ground, crawling, touching objects, and putting their fingers, toys, and other objects in their mouths), and the fact that their brains and organs are still developing (Eskenazi et al., 1999). Human and animal studies show that several mechanisms explain the effects of pesticide exposure on children, and specifically on child growth outcomes in early life. Pesticides can lead to endocrine, hormonal, thyroid, and placental disruption in pregnancy (Mustieles et al., 2017; Silvia et al., 2020; Chevrier et al., 2008; Milczarek et al., 2016; Cecchi et al., 2021) and to thyroid disruption in neonates (Sun et al., 2022). Pesticides can also influence glucose metabolism (Debost-Legrand et al., 2016), immune regulation (Neta et al., 2011; Tyagi et al., 2016), and the bacterial composition of the gut microbiome (Yuan et al., 2019) as well as contribute to environmental enteric dysfunction (EED), a subclinical condition resulting in increased gut permeability and impaired nutrient absorption (Mapesa et al., 2016).

Studies conducted in high-income countries (primarily the United States) have consistently shown that prenatal exposure to organochlorines is associated with adverse birth outcomes, including preterm birth, small-for-gestational age, smaller head circumference, and shorter femur length (Longnecker et al., 2001; Ouidir et al., 2020). In contrast, evidence suggests that prenatal exposure to organophosphate insecticides and herbicides is not associated with adverse birth outcomes (Reiss et al., 2015; Shirangi et al., 2011; de Araujo et al., 2016). With respect to associations between prenatal pesticide exposure and birth weight and length, evidence is less conclusive. Some studies have linked prenatal exposure to organochlorines with increased birth weight, overweight and elevated body mass index (BMI) in infancy and childhood (Mendez et al., 2011; Pinos et al., 2021; Stratakis et al., 2022), while other studies have shown that prenatal organochlorine exposure is associated with lower birth weight (Wolff et al., 2007; Govarts et al., 2012). Similarly, the literature reports no consistent associations of organochlorines and birth length (Wolff et al., 2007; Kalloo et al., 2020). With respect to organophosphates, a recent meta-analysis which pooled 10 studies (eight from high-income countries and two from LMICs) found that prenatal organophosphate exposure was not associated with birth weight (Khoshhali et al., 2020). Although some additional studies have shown null associations between prenatal organophosphate exposure and birth weight, corroborating the conclusion from this meta-analysis (Balalian et al., 2021), others have shown that prenatal exposure to organophosphates is associated with lower birth weight (Rauch et al., 2012; Gemmill et al., 2013). Whether prenatal organophosphate exposure is associated with birth length remains unclear as well, with studies showing positive (Eskenazi et al., 2004), negative (Rauch et al., 2012; Gemmill et al., 2013), and the null (Khoshhali et al., 2020) associations with birth length.

Though more limited, evidence on postnatal exposure suggests that postnatal pesticide exposure can also be harmful for child growth. Another recent systematic review assessing prenatal and postnatal pesticide exposure and obesity in children included eight studies of postnatal pesticide exposure (five in high-income countries and three in LMICs) (Pinos et al., 2021). This systematic review found that postnatal exposure to organophosphates was associated with higher BMI Z-scores in boys at four years of age and higher waist circumference and obesity in children 6–18 years of age. Postnatal exposure to organochlorines was not associated with weight gain during childhood and adolescence, but was associated with lower BMI Z-score in boys (Pinos et al., 2021). Further, evidence suggests that the effects of prenatal pesticide exposure may manifest later in infancy and childhood. One pooled analysis of seven European cohorts showed that prenatal exposure to organochlorines was associated with increased child growth between birth and 24 months of age (Iszatt et al., 2015), whereas one systematic review showed that prenatal exposure to endocrine-disrupting chemicals (including organochlorines) was associated with obesity later in life (Tang-Péronard et al., 2011).

In contrast to this large body of evidence in high-income countries, less is known about the relationship between prenatal and postnatal pesticide exposure and child growth in LMICs. Therefore, the research question we aimed to answer was: *what is the effect of pesticide exposure on child growth in children <5 years of age living in LMICs?* With this objective in mind, we systematically reviewed and synthesised the evidence on the associations between pesticide exposure and adverse birth outcomes and/or impaired postnatal growth in children up to 5 years of age in LMICs. Given the limited evidence from LMICs, we kept the review broad: considering any active ingredients or mixtures of active ingredients and a wide range of child growth outcomes at birth and postnatally.

## 2 Methods

### 2.1 Search strategy, eligibility criteria, and selection process

This systematic review was conducted in accordance with the Preferred Reporting Items for Systematic Reviews and Meta-Analyses (PRISMA) Guidelines (Moher et al., 2009). It was preregistered with PROSPERO prior to commencing (CRD42021292919). The PECO (populations, exposures, comparators, and outcomes) statement for this review was (Morgan et al., 2018):

- Population: children <5 years of age in LMICs.
- Exposure: self-reported or measured prenatal (at any time during pregnancy) or postnatal exposure to pesticides.
- Comparator: a less exposed reference population (continuously, high/low exposure groups, or exposed/unexposed groups).
- Outcomes: any anthropometric measures of child linear or ponderal growth, and/or any of six birth outcomes (birth weight, birth length, low birth weight, preterm birth, small-for-gestational age, large-for-gestational age).

We searched 10 electronic databases from inception to November 2021, with no language restriction: PubMed, Cochrane Library, Embase, Scopus, LILACS, Web of Science, CAB abstracts, Global Health (CABI), Global Index Medicus, and SciELO. The selection of databases was informed by databases searched in prior systematic reviews on pesticide exposure and child neurodevelopment (Sapbamrer and Hongsibsong, 2019; González-Alzaga et al., 2014; Muñoz-Quezada et al., 2013) and through consultations with a research librarian. The inclusion of 10 databases was to ensure our review was extensive and comprehensive. Search terms included medical subject headings (MeSH), keywords, and free text words along the following themes: child, pesticides, child growth, and birth outcomes. Search terms were combined using the Boolean operators “AND” and “OR”. The search strategy was developed by LB and LMJ. The search strategy was informed by search terms and keywords used in prior systematic reviews on pesticide exposure and child neurodevelopment (Sapbamrer and Hongsibsong, 2019; González-Alzaga et al., 2014; Muñoz-Quezada et al., 2013) and through consultations with a research librarian. The complete search strategy as executed in each database is available in Supplemental Table 1. All databases were searched on December 1, 2021. Searched fields were title, abstract or author keywords. Reference lists of extracted articles were reviewed for any additional studies that may have been missed by the search.

Peer-reviewed articles were included if they met the following criteria: (1) conducted in a LMIC (based on The World Bank categorisation at the time the study was conducted); (2) assessed children <5 years of age; (3) evaluated self-reported or measured prenatal (at any time during pregnancy) or postnatal exposure to pesticides; (4) measured at least one anthropometric measure of child linear or ponderal growth, and/or one of six birth outcomes (see section 2.2). We included prospective cohort studies and cross-sectional studies, and excluded animal studies, case-control studies, simulation studies, case reports, case studies, opinions, editorials, commentaries, letters, conference abstracts, ecological studies, reviews, and systematic reviews. We also excluded studies conducted in high-income countries, assessing children >5 years of age, and focusing solely on insecticide-treated bednets for malaria prevention.

Two reviewers (LB and LMJ) independently screened all titles and abstracts for inclusion using Covidence (n.d). Disagreements were resolved through discussion, and input from a third reviewer (AR) as needed. Full texts of eligible studies were reviewed for inclusion and data extraction. One reviewer (LB) extracted information on: (1) publication details (author names, journal, year of publication), (2) study meta-data (location, setting, design, population, year(s) of data collection, duration of study, inclusion and exclusion criteria, funding source); (3) participant characteristics (number, age, sex, education, occupation); (4) pesticide exposure (types of pesticides, method of exposure assessment, time point of exposure assessment); (5) outcomes; and (6) methodological quality (study type, size, confounding and attempts to correct for confounding, list of confounding factors; and funding sources). Data extraction was reviewed by two additional reviewers (AR and LMJ). Any disagreements regarding the extracted data were resolved through discussion between the three reviewers (LB, AR, and LMJ). When information was unclear or unavailable, we did not attempt to contact the authors of the original study.

### 2.2 Outcome measures

The primary outcomes were any anthropometric measures of child linear or ponderal growth measured at any time point up to 5 years of age: length/height-for-age Z-score, weight-for-age Z-score, weight-for-length/height Z-score, BMI Z-score, and stunting, underweight, wasting, and overweight as defined by individual articles. The secondary outcomes were six birth outcomes: birth weight, birth length, low birth weight, preterm birth, small-for-gestational age, and large-for-gestational age as defined by individual articles.

### 2.3 Data analysis

Characteristics of the studies included in the review were summarised narratively by exposure characteristics (types of pesticides, method of exposure assessment, time point of exposure assessment), types of outcomes assessed, and study characteristics (study design, study year, region). Data were summarised narratively using two groupings: outcomes assessed and pesticides assessed. First, we grouped studies by the frequency of outcomes they assessed. Then, by outcome, we grouped studies by the frequency of pesticide types they assessed: organochlorines, organophosphates, pyrethroids, carbamates, and chlorophenols (non-specific biomarkers of organochlorines). Where both unadjusted and adjusted estimates were reported, we summarised evidence on adjusted estimates. For continuous outcomes, we reported mean differences (MD) and standardised mean differences (SMD). For binary outcomes, we reported odds ratios (ORs), relative risks (RR), and proportions in exposed and unexposed groups. We were unable to extract all necessary information to convert and standardise effect estimates across articles. We therefore reported effect estimates as reported by individual articles. Meta-analysis was not conducted due to the heterogeneity of studies: <2 eligible studies were conducted on the same population, assessed the same pesticides, assessed exposure over the same period, and included the same outcomes.

### 2.4 Risk of bias and quality of evidence assessment

Two reviewers (LB and LMJ) independently assessed the study risk of bias and quality of the evidence for outcomes reported in ≥ 4 studies using the GRADE approach (GRADE Working Group, 2013). The GRADE approach rates the certainty of the evidence for a given outcome based on five criteria: risk of bias, inconsistency, indirectness, imprecision, and other considerations (publication bias, large effect, plausible confounding, and dose-response gradient). The risk of bias assessment was first conducted at the study-level and then at the outcome-level. Under each criterion, the quality of the evidence is considered “not serious” (no study limitations) as a baseline, and can then be downgraded by one level to “serious” or two levels to “very serious” depending on the study limitations (GRADE Working Group, 2013). One limitation of the GRADE approach is that observational studies start as “low-quality evidence” (Guyatt et al., 2011). However, not all observational studies are of low-quality (Viswanathan et al., 2018). Given these considerations and because all the included studies in this review were observational, we assigned an initial risk of bias rating of “not serious” to all studies. We then downgraded studies for limitations specific to observational studies: failure to develop and apply appropriate eligibility criteria, flawed measurement of exposure and outcome, failure to adequately control for confounding, and incomplete follow-up (Guyatt et al., 2011). No other modifications were made to the GRADE approach. Disagreements were resolved through discussion and input from a third reviewer (AR) as needed. We used the GRADEpro Software to conduct the GRADE assessment and to prepare the “Summary of findings” table (GRADEpro GDT, 2021).

## 3 Results

### 3.1 Study selection

**Fig. 1:**
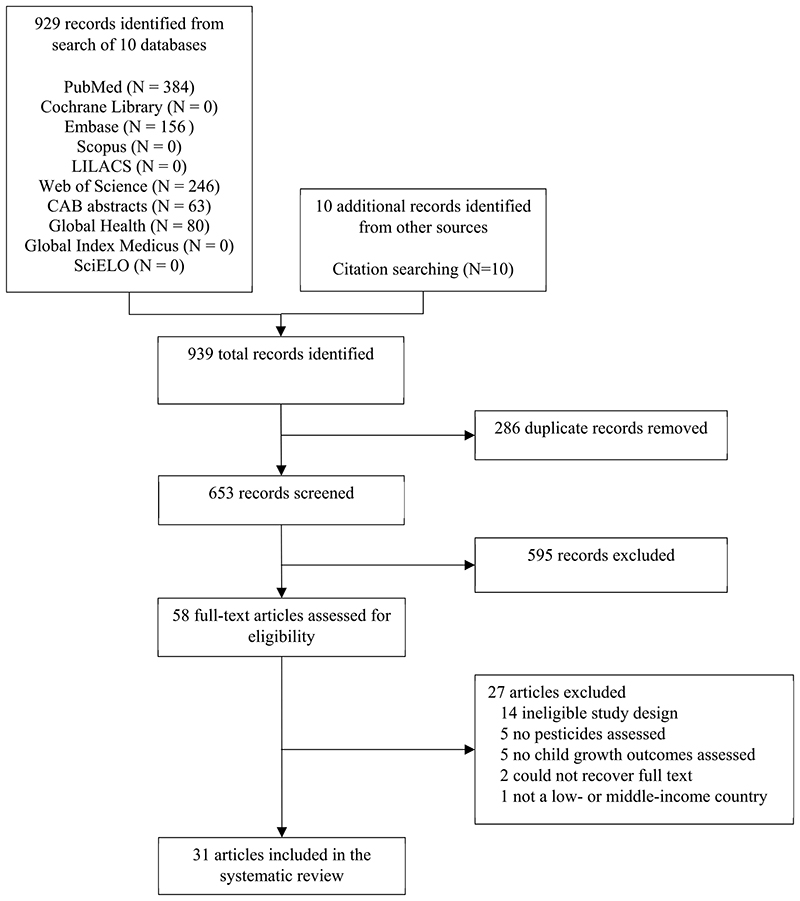
Preferred Reporting Items for Systematic Reviews and Meta-analyses (PRISMA) flow diagram of search results and included articles.

A total of 939 records were identified: 929 from the search strategy and 10 from other sources (Fig. 1). After removing the duplicates (286 records), we excluded 595 records based on title and abstract review. Proportionate agreement was 91%, with the third reviewer intervening on 9% of studies. The full texts of the remaining 58 records were reviewed and 27 were excluded. We included 31 articles in this systematic review.

### 3.2 Study characteristics

Table 1 presents the characteristics of the 31 studies included in the review. Studies were conducted in 16 countries across Asia, Central America, South America, and North Africa. The most represented countries were China (n = 11) (Ding et al., 2015; Fang et al., 2019; Zhang et al., 2018; Guo et al., 2014, 2016; Wang et al., 2012a, 2012b; Xu et al., 2017; Yang et al., 2020, 2021; Liu et al., 2016), Mexico (n = 3) (Cupul-Uicab et al., 2010; Moreno-Banda et al., 2009; Garced et al., 2012), Argentina (n = 2) (Silvia et al., 2020; Cecchi et al., 2021), India (n = 2) (Anand and Taneja, 2020; Dwivedi et al., 2022), and Poland (n = 2) (Jurewicz et al., 2005; Hanke et al., 2003). Studies were published between 2003 and 2022. Eleven were prospective cohort studies (Cecchi et al., 2021; Ding et al., 2015; Fang et al., 2019; Zhang et al., 2018; Yang et al., 2021; Liu et al., 2016; Garced et al., 2012; Naksen et al., 2015; Toichuev et al., 2018; Jaacks et al., 2019; Arrebola et al., 2016) and 20 cross-sectional studies (Silvia et al., 2020; Guo et al., 2014, 2016; Wang et al., 2012a, 2012b; Xu et al., 2017; Yang et al., 2020; Cupul-Uicab et al., 2010; Moreno-Banda et al., 2009; Anand and Taneja, 2020; Dwivedi et al., 2022; Jurewicz et al., 2005; Hanke et al., 2003; Steinholt et al., 2020; Abdel Hamid et al., 2020; Rahimi et al., 2020; Cioroiu et al., 2010; Bravo et al., 2019; Gladen et al., 2003; Van Tung et al., 2016). Analytic sample sizes ranged from 52 to 1100 (n = 11,991 total participants). Most articles (23 out of 31, 74%) assessed biomarkers in urine (n = 8) (Ding et al., 2015; Zhang et al., 2018; Guo et al., 2016; Wang et al., 2012b; Liu et al., 2016; Naksen et al., 2015; Jaacks et al., 2019; Steinholt et al., 2020), blood (n = 12) (Fang et al., 2019; Guo et al., 2014; Xu et al., 2017; Yang et al., 2020, 2021; Cupul-Uicab et al., 2010; Garced et al., 2012; Dwivedi et al., 2022; Arrebola et al., 2016; Steinholt et al., 2020; Abdel Hamid et al., 2020; Bravo et al., 2019), placenta (n = 2) (Anand and Taneja, 2020; Toichuev et al., 2018), and breastmilk (n = 2) (Gladen et al., 2003; Van Tung et al., 2016). The remaining eight articles (26%) used self-reported exposure (Silvia et al., 2020; Cecchi et al., 2021; Wang et al., 2012a, 2012b; Moreno-Banda et al., 2009; Jurewicz et al., 2005; Hanke et al., 2003; Rahimi et al., 2020). All 31 articles assessed prenatal exposure. Four studies reported outcomes beyond birth (Yang et al., 2021; Cupul-Uicab et al., 2010; Jaacks et al., 2019; Van Tung et al., 2016).

Five out of the 31 studies assessed multiple pesticide types (Cecchi et al., 2021; Yang et al., 2020; Jurewicz et al., 2005; Hanke et al., 2003; Jaacks et al., 2019). The most analysed pesticides were organochlorines or their metabolites (17 out of 31 studies, 55%), including dichlorodiphenyltrichloroethane (DDT) and its metabolites, hexachlorobenzene (HCB), hexachlorocyclohexane (HCH) and its isomers, mirex, endosulfan, aldrin, dieldrin, endrin aldehyde, trans-nonachlor, oxychlordane, and heptachlor epoxide (Fang et al., 2019; Guo et al., 2014; Xu et al., 2017; Yang et al., 2021; Cupul-Uicab et al., 2010; Garced et al., 2012; Anand and Taneja, 2020; Dwivedi et al., 2022; Toichuev et al., 2018; Arrebola et al., 2016; Steinholt et al., 2020; Abdel Hamid et al., 2020; Cioroiu et al., 2010; Bravo et al., 2019; Gladen et al., 2003; Van Tung et al., 2016; Bixby et al., 2019). Organophosphates or their metabolites were measured in 29% of studies (9 out of 31) (Cecchi et al., 2021; Wang et al., 2012b; Yang et al., 2020; Liu et al., 2016; Moreno-Banda et al., 2009; Jurewicz et al., 2005; Hanke et al., 2003; Naksen et al., 2015; Jaacks et al., 2019), pyrethroids in 13% of studies (4 out of 31) (Ding et al., 2015; Jurewicz et al., 2005; Hanke et al., 2003; Jaacks et al., 2019), and carbamates in 13% of studies (4 out of 31) (Cecchi et al., 2021; Zhang et al., 2018; Yang et al., 2020; Hanke et al., 2003). One study assessed chlorophenols, non-specific biomarkers of organochlorines (Guo et al., 2016).

Four of the eight studies assessing self-reported exposure did not specify the types of pesticides used (Cecchi et al., 2021; Wang et al., 2012a; Moreno-Banda et al., 2009; Rahimi et al., 2020). One study assessed both self-reported pesticide exposure and urinary metabolites, and did not specify the types of pesticides used based on self-report (Wang et al., 2012b). One study collected blood samples but used them to assess acetylcholinesterase and butyrylcholinesterase activity and hormone levels. The types of pesticides used were specified as in secticides of the organophosphate and carbamate families, based on commonly used pesticides (Silvia et al., 2020). The remaining two studies were conducted in Poland, which was an LMIC at the time the studies were conducted. In one of these studies, which assessed residential exposure, the most frequently reported pesticides were organophosphorus compounds, pyrethroids, phenoxyacetic acid derivatives, and benzene thiosulphonate derivatives (Hanke et al., 2003). In the second study, which assessed occupational exposure at greenhouses, those responsible for chemical protection at the greenhouses reported the use of 17 pesticides primarily used as insecticides, fungicides, and acaricides (Jurewicz et al., 2005).

The most frequently analysed outcome was birth weight (24 out of 31 studies, 77%) (Silvia et al., 2020; Cecchi et al., 2021; Ding et al., 2015; Fang et al., 2019; Zhang et al., 2018; Guo et al., 2014, 2016; Wang et al., 2012a, 2012b; Xu et al., 2017; Yang et al., 2020; Liu et al., 2016; Anand and Taneja, 2020; Dwivedi et al., 2022; Jurewicz et al., 2005; Hanke et al., 2003; Naksen et al., 2015; Jaacks et al., 2019; Arrebola et al., 2016; Steinholt et al., 2020; Abdel Hamid et al., 2020; Bravo et al., 2019; Gladen et al., 2003; Van Tung et al., 2016), followed by birth length (15 out of 31 studies, 48%) (Silvia et al., 2020; Cecchi et al., 2021; Ding et al., 2015; Fang et al., 2019; Zhang et al., 2018; Guo et al., 2016; Wang et al., 2012b; Xu et al., 2017; Liu et al., 2016; Anand and Taneja, 2020; Naksen et al., 2015; Arrebola et al., 2016; Steinholt et al., 2020; Abdel Hamid et al., 2020; Bravo et al., 2019), low birth weight (nine out of 31 studies, 29%) (Silvia et al., 2020; Cecchi et al., 2021; Wang et al., 2012a; Moreno-Banda et al., 2009; Jurewicz et al., 2005; Toichuev et al., 2018; Jaacks et al., 2019; Rahimi et al., 2020; Van Tung et al., 2016), and preterm birth (six out of 31 studies, 19%) (Silvia et al., 2020; Cecchi et al., 2021; Jurewicz et al., 2005; Jaacks et al., 2019; Rahimi et al., 2020; Cioroiu et al., 2010). Birth weight was directly assessed in 10 studies (out of 24, 42%) (Cecchi et al., 2021; Fang et al., 2019; Guo et al., 2016; Anand and Taneja, 2020; Dwivedi et al., 2022; Jaacks et al., 2019; Arrebola et al., 2016; Abdel Hamid et al., 2020; Bravo et al., 2019; Van Tung et al., 2016), based on maternal report in one study (4%) (Jurewicz et al., 2005), and based on medical records in 11 studies (46%) (Ding et al., 2015; Zhang et al., 2018; Guo et al., 2014; Wang et al., 2012a, 2012b; Yang et al., 2020; Liu et al., 2016; Hanke et al., 2003; Naksen et al., 2015; Steinholt et al., 2020; Gladen et al., 2003). Two studies (out of 24, 8%) did not specify how birth weight was assessed (Silvia et al., 2020; Xu et al., 2017). Birth length was directly assessed in seven studies (out of 15, 47%) (Cecchi et al., 2021; Fang et al., 2019; Guo et al., 2016; Anand and Taneja, 2020; Arrebola et al., 2016; Abdel Hamid et al., 2020; Bravo et al., 2019) and based on medical records in six studies (40%) (Ding et al., 2015; Zhang et al., 2018; Wang et al., 2012b; Liu et al., 2016; Naksen et al., 2015; Steinholt et al., 2020). Two studies (out of 15, 13%) did not specify how birth weight was assessed (Silvia et al., 2020; Xu et al., 2017). All nine studies that assessed low birth weight defined it as birth weight <2500 g (Cecchi et al., 2021; Wang et al., 2012a; Moreno-Banda et al., 2009; Jurewicz et al., 2005; Toichuev et al., 2018; Jaacks et al., 2019; Rahimi et al., 2020; Van Tung et al., 2016). In these studies, birth weight was directly assessed (n = 4) (Cecchi et al., 2021; Wang et al., 2012b; Jaacks et al., 2019; Van Tung et al., 2016), based on maternal report (n = 2) (Moreno-Banda et al., 2009; Jurewicz et al., 2005), based on medical records (n = 2) (Moreno-Banda et al., 2009; Toichuev et al., 2018), or the method was not reported (n = 2) (Silvia et al., 2020; Rahimi et al., 2020). All six studies assessing preterm birth defined it as birth before 37 weeks of gestation. Gestational age was measured by ultrasound (n = 2) (Cecchi et al., 2021; Jaacks et al., 2019), date of last menstrual period (n = 1) (Cecchi et al., 2021), or maternal self-report (n = 2) (Jurewicz et al., 2005; Rahimi et al., 2020). Two studies did not specify how gestational age was determined (Silvia et al., 2020; Cioroiu et al., 2010).

Small-for-gestational age was reported in three studies (Silvia et al., 2020; Cecchi et al., 2021; Jaacks et al., 2019), which defined it as weight-for-gestational age percentile <10th percentile based on the Intergrowth-21st standards (Cecchi et al., 2021; Jaacks et al., 2019) or on national standards (Silvia et al., 2020). The following outcomes were reported in two studies: BMI Z-score (Yang et al., 2021; Garced et al., 2012), height (Cupul-Uicab et al., 2010; Van Tung et al., 2016), length-for-age Z-score (Garced et al., 2012; Jaacks et al., 2019), weight-for-age Z-score (Garced et al., 2012; Jaacks et al., 2019), and weight-for-length Z-score (Garced et al., 2012; Jaacks et al., 2019). Z-scores were based on the World Health Organisation (WHO) 2006 Child Growth Standards (WHO Multicentre Growth Reference Study Group, 2006) in the three articles that calculated them (Yang et al., 2021; Garced et al., 2012; Jaacks et al., 2019). The following outcomes were reported in only one study: BMI (Cupul-Uicab et al., 2010); large-for-gestational age, defined as weight-for-gestational age percentile >90th percentile based on the Intergrowth-21st standards (Cecchi et al., 2021); overweight, defined as BMI Z-score >85th percentile of the WHO standards (Yang et al., 2021); stunting, defined as length-for-age Z-score < -2 SD based on the WHO standards (Jaacks et al., 2019); term low birth weight (Jurewicz et al., 2005), and weight (Van Tung et al., 2016).

### 3.3 Study risk of bias assessment

Risk of bias was “not serious” for most studies (22 out of 31 studies, 71%) (Table 1). Risk of bias was downgraded to “serious” for the reaming nine studies (29%) due to no/inadequate attempts to correct for confounding and potential bias in the measurement of exposure.

### 3.4 Associations with birth weight

Of the 24 articles reporting on birth weight, 12 assessed associations with organochlorines (Fang et al., 2019; Guo et al., 2014; Xu et al., 2017; Yang et al., 2020; Anand and Taneja, 2020; Dwivedi et al., 2022; Arrebola et al., 2016; Steinholt et al., 2020; Abdel Hamid et al., 2020; Bravo et al., 2019; Gladen et al., 2003; Van Tung et al., 2016), nine with organophosphates (Silvia et al., 2020; Cecchi et al., 2021; Wang et al., 2012b; Yang et al., 2020; Liu et al., 2016; Jurewicz et al., 2005; Hanke et al., 2003; Naksen et al., 2015; Jaacks et al., 2019), three with pyrethroids (Ding et al., 2015; Hanke et al., 2003; Jaacks et al., 2019), five with carbamates (Silvia et al., 2020; Cecchi et al., 2021; Zhang et al., 2018; Yang et al., 2020; Hanke et al., 2003), one reporting on occupational exposure did not specify the types of pesticides used (Wang et al., 2012a), and one assessed chlorophenols non-specific to organochlorines (Guo et al., 2016).

Of the 12 studies that assessed associations between organochlorines and birth weight, nine found significant associations. Four studies found significant positive associations with DDT, DDE, and DDD ranging from 0.008 to 0.25 SMD (Xu et al., 2017; Arrebola et al., 2016; Steinholt et al., 2020; Bravo et al., 2019). One study found a significant negative association with total DDT of 0.009 g (Anand and Taneja, 2020), and two studies found significant negative associations with HCH and its isomers ranging from –5.81 to –55.14 g (Yang et al., 2020; Anand and Taneja, 2020) (Table 2). One study showed that higher concentrations of α-HCH, DDE isomers, and dieldrin were significantly correlated with lower birth weight (Dwivedi et al., 2022). Another study observed significantly different birth weight by tertile of prenatal concentrations of β-HCH, but no indication of dose response (Gladen et al., 2003). Three studies found no significant associations between prenatal organochlorine exposure and birth weight (Fang et al., 2019; Guo et al., 2014; Abdel Hamid et al., 2020; Van Tung et al., 2016). Four of these 12 studies which assessed the associations between organochlorines and birth weight controlled for maternal BMI at the time of assessment (Guo et al., 2014; Xu et al., 2017; Arrebola et al., 2016; Steinholt et al., 2020), three controlled for pre-pregnancy BMI (Fang et al., 2019; Yang et al., 2020; Gladen et al., 2003), and none controlled for dietary intake.

Of the nine studies that assessed associations between organophosphates and birth weight, one found a significant negative association of 170 ± 60 g with higher concentrations of 4-nitrophenol (a metabolite of parathion and methyl parathion) comparing quartile 3 to quartile 1, but no significant associations with 3,5,6-trichloro-2-pyridinol (TCPY, a metabolite of chlorpyrifos and chlorpyrifos methyl) or 2-isopropyl-4-methyl-6-hydroxypyrimidine (IMPY, a diazinon metabolite) (Jaacks et al., 2019). None of the remaining eight studies found significant associations between prenatal organophosphate exposure and birth weight (Silvia et al., 2020; Cecchi et al., 2021; Wang et al., 2012b; Yang et al., 2020; Liu et al., 2016; Jurewicz et al., 2005; Hanke et al., 2003; Naksen et al., 2015) (Table 2).

Two of the studies that assessed associations between pyrethroids and birth weight found significant negative associations ranging from 96.76 to 233.3 g (Ding et al., 2015; Hanke et al., 2003) and two found no significant associations (Jurewicz et al., 2005; Jaacks et al., 2019) (Table 2). None of the five studies that assessed associations between carbamates and birth weight found significant associations (Silvia et al., 2020; Cecchi et al., 2021; Zhang et al., 2018; Yang et al., 2020; Hanke et al., 2003). The study that assessed chlorophenols non-specific to organochlorines found that higher concentrations of 2,4,6-trichlorophenol (TCP) and pentachlorophenol (PCP) were associated with –30 g and –37 g lower birth weight, respectively (Guo et al., 2016) (Table 2).

### 3.5 Associations with birth length

Of the 15 articles reporting on birth length, seven assessed associations with organochlorines (Fang et al., 2019; Xu et al., 2017; Anand and Taneja, 2020; Arrebola et al., 2016; Steinholt et al., 2020; Abdel Hamid et al., 2020; Bravo et al., 2019), five with organophosphates (Silvia et al., 2020; Cecchi et al., 2021; Wang et al., 2012b; Liu et al., 2016; Naksen et al., 2015), one with pyrethroids (Ding et al., 2015), three with carbamates (Silvia et al., 2020; Cecchi et al., 2021; Zhang et al., 2018), and one assessed chlorophenols non-specific to organochlorines (Guo et al., 2016).

Of the seven studies that assessed the associations between organochlorines and birth length, one study found a significant positive association of 0.21 SMD with higher levels of p,p′-DDT (Bravo et al., 2019). Another study found significant negative associations with o,p′-DDE and p,p′-DDD ranging from 0.25 to 0.32 SMD and a 0.25 SMD significant positive association with aldrin (Steinholt et al., 2020). The remaining five studies found no significant associations between prenatal exposure to organochlorines and birth length (Fang et al., 2019; Xu et al., 2017; Anand and Taneja, 2020; Arrebola et al., 2016; Abdel Hamid et al., 2020).

Four of the five studies that assessed prenatal organophosphate exposure found no significant associations with birth length (Silvia et al., 2020; Wang et al., 2012b; Liu et al., 2016; Naksen et al., 2015). One study found 0.372 cm lower birth length among those in residential proximity to fruit croplands (where organophosphates and carbamates were frequently used) relative to those not in residential proximity to fruit croplands (Cecchi et al., 2021). Another study found no association between prenatal carbofuranphenol levels and birth length (Zhang et al., 2018). The one study that assessed associations between pyrethroid metabolites and birth length found no significant associations (Ding et al., 2015). The study that assessed chlorophenols non-specific to organochlorines also found no significant associations with birth length (Guo et al., 2016).

### 3.6 Associations with low birth weight

Of the nine articles reporting on low birth weight, two assessed associations with organochlorines (Toichuev et al., 2018; Van Tung et al., 2016), five with organophosphates (Silvia et al., 2020; Cecchi et al., 2021; Moreno-Banda et al., 2009; Jurewicz et al., 2005; Jaacks et al., 2019), two with pyrethroids (Jurewicz et al., 2005; Jaacks et al., 2019), two with carbamates (Silvia et al., 2020; Cecchi et al., 2021), and two reporting on occupational exposure did not specify the types of pesticides used (Wang et al., 2012a; Rahimi et al., 2020).

None of the studies that assessed the associations between organochlorines or pyrethroids and low birth weight found significant associations (Jurewicz et al., 2005; Toichuev et al., 2018; Jaacks et al., 2019; Van Tung et al., 2016). Of the five studies that assessed associations with organophosphates, one found 2.13 (95% CI 1.12, 4.08) higher risk of low birth weight among women with detectable prenatal levels of IMPY relative to women with undetectable levels (Jaacks et al., 2019). Another study reporting on residential exposure to organophosphates and carbamates found that 6% of women assessed during the spraying season had low birth weight infants whereas 0% of women assessed during the non-spraying season had low birth weight infants (Silvia et al., 2020). The remaining studies assessing organophosphates or carbamates found no significant associations between prenatal exposure and low birth weight (Cecchi et al., 2021; Moreno-Banda et al., 2009; Jurewicz et al., 2005). One of the studies reporting on occupational exposure found significantly higher prevalence of low birth weight (11.6%) among greenhouse workers compared to housewives (3.2%) (Rahimi et al., 2020). However, the second study found no significant associations between greenhouse work during pregnancy and birth length (Rahimi et al., 2020).

### 3.7 Associations with pre-term birth

Of the six studies reporting on preterm birth, one assessed associations with organochlorines (Cioroiu et al., 2010), four with organophosphates (Silvia et al., 2020; Cecchi et al., 2021; Jurewicz et al., 2005; Jaacks et al., 2019), two with pyrethroids (Jurewicz et al., 2005; Jaacks et al., 2019), two with carbamates (Silvia et al., 2020; Cecchi et al., 2021), and one reporting on occupational exposure did not specify the types of pesticides used (Rahimi et al., 2020).

The study assessing associations between organochlorines and preterm birth found significantly higher prenatal levels of total DDT in women with preterm births (715 ± 952 ng/g) than in women with normal births (687 ± 1393 ng/g), but lower prenatal levels of total HCH in women with preterm births (174 ± 210 ng/g) than in women with normal births (207 ± 337 ng/g) (Cioroiu et al., 2010). One of the four studies assessing associations with organophosphates found significantly higher risk of preterm birth among women with higher prenatal concentrations of 4-nitrophenol: RR 3.57 (95% CI 1.65, 7.73) comparing quartile 3 to quartile 1, RR 3.57 (95% CI 1.65, 7.73) comparing quartile 4 to quartile 1; and RR 1.44 (95% CI 1.17, 1.78) per μg/g creatinine adjusted higher 4-nitrophenol (Jaacks et al., 2019). Another study reporting on residential exposure to organophosphates and carbamates found that 6% of women assessed during the spraying season had preterm births whereas 0% of women assessed during the non-spraying season had preterm births (Silvia et al., 2020). The remaining studies assessing organophosphates, pyrethroids, or carbamates found no significant associations between prenatal exposure and preterm birth (Cecchi et al., 2021; Jurewicz et al., 2005; Jaacks et al., 2019; Rahimi et al., 2020).

### 3.8 Associations with other outcomes

Of the three studies that assessed associations with small-for-gestational age, one found that higher prenatal concentrations of 4-nitrophenol were associated with 4-times higher risk of a small-for-gestational age birth, whereas higher prenatal concentrations of TCPY and 3-PBA were associated with 0.12–0.75 times significantly lower risk of a small-for-gestational age birth, respectively (Jaacks et al., 2019). The second found that residential proximity to fruit croplands during pregnancy was not associated with either small- or large-for-gestational age births (Cecchi et al., 2021). The third study found no small-for-gestational births either among women assessed during the spraying season or those assessed during the non-spraying season (Silvia et al., 2020). With respect to anthropometry at birth, one study showed that higher concentrations of p,p’-DDE in each trimester of pregnancy were not associated with BMI Z-score, length-for-age Z-score, weight-for-age Z-score, or weight-for-length Z-score (Garced et al., 2012). Lastly, one study found that working in a greenhouse was not associated with low birth weight in term pregnancies (Jurewicz et al., 2005).

### 3.9 Associations with outcomes later in infancy

Of the four studies that reported outcomes later in infancy, two studies found that prenatal exposure to organochlorines was not associated with child height or weight at 8–9 or 12–14 weeks of age (Van Tung et al., 2016), or with child BMI or height at 16 months of age (Cupul-Uicab et al., 2010). One study found that higher prenatal concentrations of the organophosphate 4-nitrophenol were significantly associated with lower length-for-age Z-score (–0.50 ± 0.25 SD comparing quantile 3 to quantile 1) and lower weight-for-age Z-score (with an increasing dose response relationship with higher concentrations ranging from –0.35 ± 0.17 to –0.66 0.17 SD) at 1 year of age (Jaacks et al., 2019). Prenatal levels of 4-nitrophenol showed no consistent associations with weight-for-length Z-score at 1 year of age with a significant positive association comparing quantile 2 to quantile 1, but a negative association comparing quantile 4 to quantile 1. At 2 years of age, higher prenatal concentrations of the organophosphate metabolite TCPY were associated with significantly lower length-for-age Z-score and higher prenatal concentrations of 4-nitophenol were significantly associated with lower weight-for-length Z-score. Prenatal pesticide exposure was not associated with stunting at 1 or 2 years of age in that study (Jaacks et al., 2019). Finally, one study found that higher prenatal exposure to a p,p′-DDE and total DDT were associated with significantly lower BMI Z-score at 6 months of age, while higher prenatal exposure to p,p′-DDT was associated with higher BMI Z-score at 12 months of age (Yang et al., 2021). In contrast, higher prenatal concentrations of HCH isomers were associated with significantly higher BMI Z-score at 6, 12, and 24 months of age (Yang et al., 2021).

### 3.10 Subgroup analysis

Of the 31 studies included in this review, six conducted subgroup analyses: five by child gender (Fang et al., 2019; Zhang et al., 2018; Guo et al., 2016; Wang et al., 2012b; Liu et al., 2016) and one by paraoxonase 1 phenotype (Naksen et al., 2015). Fang et al., 2019 found no associations between prenatal exposure to organochlorines and birth weight or length in the full sample; however, in sub-group analysis they found that higher prenatal concentrations of HCH isomers were associated with –34.48 to –28.61 g significantly lower birth weight among boys (Fang et al., 2019). Guo et al., 2016 found no association between prenatal exposure to non-specific organochlorines and birth length, but a significant negative association between higher prenatal PCP concentrations and birth length among boys 0.23 (95% CI -0.41, 0.01). Sub-group analysis also revealed that the observed associations between higher prenatal concentrations of 2,4,6-TCP and PCP and lower birth weight were only significant among boys and not girls (Guo et al., 2016). The remaining three studies found no differences in the associations between prenatal exposure to organophosphates or carbamates and birth weight and length in girls versus boys (Zhang et al., 2018; Wang et al., 2012b; Liu et al., 2016). Naksen et al., 2015 examined the associations between prenatal exposure to organophosphates and birth weight and length by paraoxonase 1 phenotype and found no differences (Naksen et al., 2015).

### 3.11 Timing of exposure assessment

Of the 31 articles included in this review, 23 assessed exposure at a single time point: 16 at delivery/birth (Ding et al., 2015; Fang et al., 2019; Zhang et al., 2018; Guo et al., 2014, 2016; Wang et al., 2012b; Xu et al., 2017; Yang et al., 2020, 2021; Liu et al., 2016; Cupul-Uicab et al., 2010; Anand and Taneja, 2020; Dwivedi et al., 2022; Toichuev et al., 2018; Abdel Hamid et al., 2020; Gladen et al., 2003), five during the third trimester of pregnancy (Silvia et al., 2020; Arrebola et al., 2016; Steinholt et al., 2020; Cioroiu et al., 2010; Bravo et al., 2019), and two at the first antenatal care visit when the pregnancy was confirmed (Hanke et al., 2003; Jaacks et al., 2019). Of the remaining eight studies, three assessed exposure once in each trimester of pregnancy (Moreno-Banda et al., 2009; Garced et al., 2012; Naksen et al., 2015) and five assessed exposure over the entire pregnancy and preconception period by using occupation or residential proximity as a proxy for exposure (Cecchi et al., 2021; Wang et al., 2012a; Jurewicz et al., 2005; Rahimi et al., 2020; Van Tung et al., 2016). None of the studies assessing exposure in each trimester of pregnancy found differences by trimester (Moreno-Banda et al., 2009; Garced et al., 2012; Naksen et al., 2015). Of the five studies using proxy measures covering pregnancy and preconception, three found no significant associations with child growth outcomes. One study found significantly lower birth length among exposed than unexposed children (-0.61 ± 1.16 vs -0.24 ± 1.04 respectively) (Cecchi et al., 2021), while another found significantly higher prevalence of preterm birth among greenhouse workers than housewives (11.9% vs 6.1%, respectively) (Rahimi et al., 2020).

### 3.12 Certainty of the evidence assessment

The certainty of the evidence was “very low” for all outcomes reported in ≥4 studies (e.g., birth weight, birth length, low birth weight, and preterm birth) due to downgrades for inconsistency, indirectness, and imprecision (Supplemental Table 2).

## 4 Discussion

In this systematic review, we sought to assess the effect of pesticide exposure on birth outcomes and/or postnatal growth in children <5 years of age living in LMICs. Although we searched 10 databases and allowed for broad definitions of the exposures and outcomes, our search strategy identified only 653 unique records, highlighting the limited literature on the topic in both high-income countries and LMICs. We included 31 studies from LMICs assessing the associations between prenatal pesticide exposure and child growth outcomes at birth or up to age 5 years. We found no consistent associations between prenatal pesticide exposure and birth weight and birth length for all pesticide classes, including associations for subgroups by child sex. Prenatal exposure to organochlorines appeared to be associated with birth weight, but the direction of this association remained unclear with studies demonstrating both positive and negative associations. Prenatal exposure to pyrethroids may be associated with lower birth weight. Similarly, findings with respect to birth length were not consistent, with positive, negative, and null associations observed in the literature. In addition, we found no consistent evidence of an association between prenatal pesticide exposure and low birth weight and preterm birth. Associations with growth outcomes later in infancy (including the primary outcomes of anthropometric measures of linear and ponderal growth) were also inconclusive given the limited number of studies. The certainty of the evidence was “very low” for all outcomes.

Our findings extend existing evidence on the associations between pesticide exposure and child growth in two key ways. First, to the best of our knowledge, this is the first systematic review to focus only on LMICs. Previous systematic reviews have included both high-income countries and LMICs without comparing and contrasting findings across these two groups (Khoshhali et al., 2020; Tang-Péronard et al., 2011). This distinction is important given that approved and commonly used pesticides vary between high-income countries and LMICs (Donley, 2019a). Second, whereas prior systematic reviews have focused on a single pesticides, e.g., glyphosate (de Araujo et al., 2016), a specific outcome, e.g., obesity (Pinos et al., 2021), or a single exposure period, e.g., prenatally (Khoshhali et al., 2020), we considered all types of pesticides, a wide range of child growth and birth outcomes, and both prenatal and postnatal exposure. Thus, we conducted a broader literature review than prior studies.

Considerable evidence shows that several mechanisms underlie the associations between pesticide exposure and child growth, including endocrine, hormonal, thyroid, and placental disruption in pregnancy (Mustieles et al., 2017; Silvia et al., 2020; Chevrier et al., 2008; Milczarek et al., 2016; Cecchi et al., 2021), thyroid disruption in the neonatal period (Sun et al., 2022), glucose metabolism disruption (Debost-Legrand et al., 2016), immune dysregulation (Neta et al., 2011; Tyagi et al., 2016), disruption of the gut microbiome (Yuan et al., 2019), and increased gut permeability and impaired nutrient absorption (Mapesa et al., 2016). Despite this considerable literature, empirical evidence on the precise mechanisms through which pesticides influence child growth in LMICs is lacking. Of the 31 studies included in this review, only one conducted mediation analysis to formally assess mechanisms and found that prenatal exposure to organochlorines reduced birth weight by disrupting thyroid hormone metabolism and glyceraldehyde metabolism (Yang et al., 2020). Two more studies assessed mechanisms without conducting mediation analysis and found that prenatal pesticide exposure was associated with more pregnancy complications, including premature rupture of membranes, intrauterine growth retardation, and threatened miscarriage (Silvia et al., 2020; Cecchi et al., 2021). More work is needed to understand how different mechanisms influence specific child growth outcomes at birth and later in infancy. Mediation models can help quantify these mechanisms, build the empirical base on mechanisms, and inform the design and targeting of interventions to reduce any potential adverse effects of prenatal pesticide exposure on child growth.

Given these plausible biological and physiological mechanisms through which pesticide exposure during pregnancy can influence child growth, the lack of consistent associations in LMICs is likely due to two important limitations of the current literature. One major limitation is the incomplete toxicology picture. Most studies included in this review assessed a few active ingredients and some did not assess specific pesticides but used residential location or occupation as a proxy for pesticide exposure. A recent study identified 239 active pesticide ingredients approved for use in China (Donley, 2019a), the country with the most studies included in this review, which evaluated a small fraction of these approved active ingredients. Moreover, people are not exposed to a single active ingredient but rather to a complex mixture of active ingredients and their metabolites. None of the studies included in this review explored combinations or complex mixtures of ingredients and thus it is not possible to disentangle the effects of individual pesticides from the effects of multiple pesticides including potential additive or synergistic effects (Pinos et al., 2021). Another limitation of the biomarkers assessed in included studies is the fact that some biomarkers are non-specific to pesticides and capture by-products of industrial processes (Guo et al., 2016). Further, most of the biomarkers assessed were developed for high-income settings (largely by the US Centers for Disease Control and Prevention) and do not match to frequently used active ingredients in LMICs. For example, one of the included studies from Bangladesh used a standard set of biomarkers used by the Centers for Disease Control and Prevention, several of which were not detected in most participants and one of which was not detected in any participants (Jaacks et al., 2019). Lastly, many included studies evaluated active ingredients such as DDT and other organochlorines which have been banned for use in agriculture in many settings including LMICs for over a decade (Donley, 2019b). Thus, levels of these pesticides are likely to reflect exposure through consumption of animal-source foods such as fatty fish, which tend to have the highest concentrations of organochlorines (Schecter et al., 2010). Despite this evidence, only one of the studies included in this review controlled for dietary intake during pregnancy in assessing the association between prenatal pesticide exposure and weight-related anthropometric Z-scores in infancy (Garced et al., 2012). Given that intake of animal-source foods is positively associated with both organochlorine pesticide exposure (Marks et al., 2021) and child growth (Asare et al., 2022), this residual confounding likely biased results towards the null. Future studies should explore additional biomarkers that reflect commonly used pesticides and active ingredients in a specific setting. This could be done by reviewing country-specific lists of approved active ingredients (Donley, 2019a) or by collecting information on pesticides sold in local markets. More accurate quantitative data on commonly used pesticides in LMICs is needed to better understand exposure levels and their associations not only with the child growth outcomes we reviewed here, but also with maternal and child perinatal health outcomes more broadly. Local capacity should also be built and strengthened to support research and address issues around infrastructure and resources to measure pesticide biomarkers in LMICs (Barnett-Itzhaki et al., 2018). Advances in exposure assessment from high-income settings (e.g., personal passive samplers) can help reduce the cost and participant burden of pesticide assessments in LMICs (Anderson et al., 2014; O’Connell et al., 2014).

The second major limitation of the existing literature is that most studies assessed pesticides at a single time point during the third trimester of pregnancy or at delivery/birth. Moreover, studies of or ganophosphates, pyrethroids, and carbamates mostly assessed biomarkers with short half-lives. Thus, most of the included studies did not capture chronic exposure levels. Chronic low-level exposure to pesticides has been linked to numerous neurologic functioning, neuro development, and growth problems in animals and adults (Eskenazi et al., 1999). However, chronic low-level exposure in pregnant women and young children has been largely overlooked and dismissed due to lack of visible clinical signs or symptoms (Hertz-Picciotto et al., 2018). In this review, only two studies conducted repeat measures of organophosphates across trimesters to measure chronic exposure during pregnancy. Therefore, evidence is still limited on the short- and long-term effects of chronic low-level exposure during pregnancy and childhood to doses that do not produce overt clinical symptoms (Costa, 2006).

Relatedly, one limitation of our methodology was the inclusion of cross-sectional studies, which do not capture chronic exposure to pesticides with short half-lives and are subject to reverse causation (i.e., the temporal relationship between the exposure and outcome cannot be established conclusively). However, of the 20 cross-sectional studies we included, only one assessed exposure to organophosphates using biomarkers with short half-lives (Wang et al., 2012b). The remaining 19 studies assessed either exposure to organochlorines (which have long half-lives) or self-reported exposure to pesticides over reference periods covering preconception and pregnancy. However, issues relating to the temporality of exposure and outcome remain. Given that changes in weight, including during pregnancy, are correlated with concentrations of fat-soluble pesticides like organochlorines (Vizcaino et al., 2014; Grimalt et al., 2022; Jansen et al., 2017), it is possible that the outcomes could have influenced exposure levels. Nevertheless, as the evidence on the associations between pesticide exposure and child growth in LMICs is still at an early stage, cross-sectional studies play an important role in generating hypotheses for further investigation.

Although included studies were limited to a single prenatal assessment, their findings do suggest that the effects of prenatal exposure to pesticides may manifest later in life. Specifically, one study found that prenatal exposure to organophosphates was associated with poor child linear and ponderal growth at two years of age (Jaacks et al., 2019), and another study found that prenatal exposure to organochlorines was associated with higher BMI from 6 to 24 months of age (Yang et al., 2021). Relatedly, none of the included studies assessed pesticides in children providing no information on concurrent exposure or on chronic or acute exposure in children. Some of the mechanisms through which pesticides may influence child growth (e.g., changes in glucose metabolism, immune dysregulation, EED, and changes in gut microbiome) may be more active and relevant for concurrent exposure within the same individual rather than for prior exposure in the mother. Future studies in LMICs should consider assessing both acute and chronic exposure before and during pregnancy as well as in infancy and early childhood (taking a life-course approach) to better understand the concurrent and chronic short-, medium-, and long-term effects of pesticide exposure on child growth.

Despite the strengths of our methodology, including a pre-registered protocol, a search strategy developed in consultation with a research librarian, the lack of language restrictions, and the inclusion of 10 databases, some limitations of the current literature are worth noting. First, we identified only three studies (out of 653 unique records) conducted in sub-Saharan Africa, none of which met our inclusion criteria. Two of these studies were conducted in South Africa; one assessed prenatal pesticide exposure in the context of insecticide-treated bednets for malaria prevention (Coker et al., 2018), whereas the other was a case report of acute chlorpyrifos poisoning during pregnancy (Solomon and Moodley, 2007). The third study reviewed the contribution of pesticides to the burden of diabetes in sub-Saharan Africa (Azandjeme et al., 2013). Further, we identified no studies in Central America, the Caribbean, or Asia Pacific. This is particularly concerning given that Central America and the Caribbean have the highest pesticide application rates in the world (FAO, 2021). Second, many of the included studies had small sample sizes or participants were recruited at a single location, limiting the generalisability of the findings. More studies in similar populations using consistent methodologies within and across LMICs are needed to better understand if, and how, pesticides influence child growth in LMICs. Third, few studies conducted subgroup analysis and the ones that did only examined child sex and paraoxonase 1 phenotype. Other sub groups may be more important to understand if, and how, the effects of pesticides on child growth vary, to identify the most vulnerable groups, and to inform the design and targeting of intervention to reduce pesticide exposure. One important area for future research may be subgroup analysis by household wealth as wealthier households may be less exposed to pesticides because they have improved water sources, can afford less contaminated foods, and are less likely to work in agriculture and be exposed occupationally, relative to poorer households (Fairburn et al., 2019).

Lastly, none of the studies included in this review examined the causal impact of pesticide exposure on child growth. More conclusive studies identifying causal impacts are needed. One approach could be to conduct randomised controlled feeding trials of organic foods. Three small studies in the United States (sample sizes ranging from 9 to 40 children) have shown that substituting conventional foods with organic foods significantly reduced pesticide biomarkers in children (Lu et al., 2008; Bradman et al., 2015; Fagan et al., 2020; Hyland et al., 2019). Another study of 20 pregnant women, also in the United States, showed that substituting conventional fruits and vegetables for organic significantly reduced pesticide exposure during pregnancy (Curl et al., 2019). However, none of these intervention studies measured birth outcomes or child growth. Moreover, to the best of our knowledge, no organic feeding trials have been conducted in LMICs either in pregnant women or in children. These types of trials have important feasibility, economic, ethical, and sustainability implications related to the availability, accessibility, and affordability of organic foods that should be addressed by any future trials in LMICs. For instance, in many LMICs obtaining “organic” certification is prohibitively expensive. Nonetheless, consumer demand for organic food is increasing around the world including in LMICs such as India (Chandra et al., 2021). Another approach could be to conduct randomised controlled trials of agroecological interventions promoting the adoption of organic, climate-smart agricultural practices. These may be more suitable for LMICs, where one-third of adults are employed in agriculture (The World Bank, 2021). To the best of our knowledge, only one such intervention in Tanzania has been evaluated to date. This nutrition-sensitive agroecological intervention improved child dietary diversity but had no effect on child growth (Santoso et al., 2021). However, this study did not assess pesticide exposure in women or children. Another upcoming trial in Andhra Pradesh, India will assess the effect of an agroecological intervention on pesticide exposure and child growth (ISRCTN Registry, 2021), and thus help build the evidence on the causal effect of reducing pesticide exposure on child growth. Nevertheless, given these challenges in assessing the causal impact of pesticides exposure on child growth, the precautionary principle can be adopted, especially with regards to highly hazardous (Food and Agriculture Organization of the United Nations and World Health Organization, 2016).

## 5 Conclusion

This systematic review of pesticide exposure and child growth among children <5 years of age living in LMICs identified 31 articles examining the associations between prenatal pesticide exposure and child growth outcomes at birth and later in infancy. The existing literature suggests no consistent associations between prenatal pesticide exposure and birth weight, birth length, low birth weight, and preterm birth. Many included studies were at serious risk of bias, and the quality of the evidence was “very low” for all outcomes. More studies with accurate quantitative data on commonly used pesticides and their metabolites are needed to better understand if, and how, pesticides influence child growth in LMICs. Ideally, these studies should capture chronic exposure to complex mixtures using consistent methodologies in comparable populations of pregnant women and children. Given the limited resources in LMICs to accurately measure pesticides in various biological matrices following gold-standard methods, capacity and infrastructure strengthening are needed to ensure the feasibility of toxicological cohort studies in LMICs. Precautionary guidelines may be needed given the potentially harmful effects of pesticide exposure on child growth.

## Supplementary Material

Appendix A. Supplementary data

## Figures and Tables

**Table 1:** Characteristics of included studies evaluating the association between pesticide exposure and child growth.

Author, year	Country	Study design	Sample size	Population assessed	Type of exposure	Method of exposure assessment	Timing of exposure assessment	Pesticides reported Outcomes assessed	Summary of findings	Confounding factors	GRADE study risk of bias
Anand and Taneja (2020)	India	Cross-sectional	90	Pregnant women who delivered in a hospital in Agra	Occupational; accidental	Placenta sample	At birth	α-HCH, β-HCH, γ-HCH, δ-HCH, total-HCH, p, p’-DDE, p,p’-DDT, p,p’-DDD, total-DDT	Birth weight, birth length	Higher prenatal concentrations of α-HCH, β-HCH, γ-HCH, total-HCH, p, p’-DDD, and total-DDT were associated with significantly lower birth weight. No significant associations between prenatal HCH and DDT and birth length.	Not reported	Serious
Arrebola et al. (2016)	Bolivia	Prospective cohort	200	Pregnant women	Not reported	Cord blood	Prenatally at last antenatal care visit before birth	o,p′-DDT and p,p′-DDE	Birth weight, birth length	Higher prenatal concentrations of α-HCH, β-HCH, γ-HCH, total-HCH, p, p’-DDD, and total-DDT were associated with significantly lower birth weight. No significant associations between prenatal HCH and DDT and birth length.	Gestational weight and age, maternal age, parity, smoking habit, BMI	Not serious
Bravo et al. (2019)	Russia	Cross-sectional	250	Pregnant women	Not reported	Blood sample	Prenatally in the last week of pregnancy	HCB, α-HCH, β-HCH, p, p′-DDE, p,p′-DDT, mirex	Birth weight, birth length	Higher prenatal concentrations of p, p’-DDT were associated with significantly higher birth weight and birth length.	Gestational age, maternal age, parity, smoking, and alcohol consumption	Not serious
Cecchi et al. (2021)	Argentina	Prospective cohort	776	Pregnant women living in a rural food production zone in northern Patagonia for at least four years but not involved in agricultural work	Residential proximity to fruit croplands with intense pesticide application	Residential location; self-reported	Prenatally	Organophosphates (e. g., chlorpyrifos, azinphos-methyl) and carbamates (e.g., carbofuran, pirimicarb) were most frequently used in the exposed area	Birth weight, birth length, low birth weight, preterm birth, small-for-gestational age	Living in an exposed area was associated with significantly lower birth length. No significant associations with birth length, PTB, LBW, or SGA.	Not accounted for	Serious
Cioroiu et al. (2010)	Romania	Cross-sectional	63	Pregnant women	Not reported	Colostrum	Prenatally in the first week after delivery	HCB, α-HCH, β-HCH, γ-HCH, δ-HCH, total-HCH, o,p’-DDE, p,p’-DDE, o,p’-DDD, p,p’-DDT, o,p’-DDT, p,p’-DDD, total-DDT,	Preterm birth	Higher colostrum concentrations of total HCH were significantly higher in women with normal births than in women with preterm births. Higher colostrum concentrations of total DDT were significantly lower in women with normal births than in women with preterm births.	Not accounted for	Serious
Cupul-Uicab et al. (2010)	Mexico	Cross-sectional	788	Pregnant women living in Tapachula, Chiapas who gave birth to boys	Not reported	Blood sample	Prenatally at birth	p,p’-DDE, DDT	Height	Higher prenatal concentrations of p, p’-DDE and DDT were not associated with child height.	Smoking habit, hospital recruitment, rural residence, gestational age, maternal height, education	Not serious
Ding et al. (2015)	China	Prospective cohort	454	Pregnant women	Occupational, food	Urine sample	Prenatally	cis-DCCA, trans-DCCA, 3-PBA, 4-fluoro-3-PBA, cis-DBCA, total pyrethroids	Birth weight, birth length	Higher prenatal concentrations of total pyrethroids were associated with significantly lower birth weight, but not with birth length.	Infant sex, parity, pre-pregnancy BMI and weight gain, maternal age, smoking habit, length of gestation, household income	Not serious
Dwivedi et al. (2022)	India	Cross-sectional	221	Pregnant women living in Uttar Pradesh	Non-occupational	Blood and cord blood samples	Prenatally	α-HCH, β-HCH, γ-HCH, p,p’-DDE, o,p’-DDE, o, p’-DDT, aldrin, dieldrin, endrin aldehyde	Birth weight	Higher prenatal blood concentrations of α-HCH, γ-HCH, p, p’-DDE, o,p’-DDE, o, p’-DDT and dieldrin were significantly negatively correlated with birth weight. Higher prenatal cord blood concentrations of p,p’-DDE, o,p’-DDT, and dieldrin were significantly negatively correlated with birth weight.	Not accounted for	Serious
Fang et al. (2019)	China	Prospective cohort	1028	Pregnant women resident of Wuhan City	Not reported	Cord serum	Prenatally at delivery	α-HCH, β-HCH, γ-HCH, total-HCH, p,p’-DDE, p,p’-DDT, p,p’-DDD, total-DDT	Birth weight, birth length	Higher prenatal concentrations of HCH and DDT isomers were not associated with birth weight or birth length in adjusted models. Higher prenatal concentrations of β-HCH and total HCH were associated with significantly lower birth weight among boys but not girls in adjusted models	Pre-pregnancy BMI and weight gain, maternal age, parity, education, smoking habit, gestational age, family income and infant sex	Not serious
Garced et al. (2012)	Mexico	Prospective cohort	253	Pregnant women living in the state of Morelos	Not reported	Blood sample	Prenatally at enrolment and each trimester visit	p,p’-DDE, DDT	Length-for-age Z-score, weight-for-age Z-score, weight-for-length Z-score, body mass index Z-score from birth to 1 year of age	Higher prenatal concentrations of p, p’-DDE were not associated with LAZ, WAZ, WHZ, or BMIZ	Age at evaluation, maternal age, height, and parity	Not serious
Gladen et al. (2003)	Ukraine	Cross-sectional	197	Pregnant women	Not reported	Breast milk	Prenatally, 4–5 days after birth	p,p’-DDT, p,p’-DDE, β-HCH, HCB, trans-nonachlor, oxychlordane, heptachlor epoxide	Birth weight	Mean birth weight was significantly different by tertile of prenatal concentrations of β-HCH with no pattern of a dose response: infants in the lowest tertile were small, infants in the middle tertile were large, and children in the upper tertile were average. Other pesticides showed no significant patterns	City, parity, maternal age, height, pre-pregnancy BMI, and infant gender	Not serious
Guo et al. (2014)	China	Cross-sectional	81	Pregnant women	Not reported	Blood and cord blood samples	Prenatally	p,p’-DDT, o,p’-DDT, p, p’-DDE, o,p’-DDE, p,p’-DDD, o,p’-DDD, α-HCH, β-HCH, γ-HCH, δ-HCH, total HCH, HCB, heptachlor, heptachlor epoxide B, α-chlordanes, γ-chlordanes, oxychlordane, endosulfan I, mirex	Birth weight	No significant associations between prenatal concentrations of examined pesticides and birth weight	Maternal age, BMI at delivery, infant gender, and gestational week	Not serious
Guo et al. (2016)	China	Cross-sectional	1100	Mother-child pairs	Not reported	Urine sample	Prenatally at birth	2,5-DCP, 2,4-DCP, 2,4,5-TCP, 2,4,6-TCP and PCP	Birth weight	Higher prenatal concentrations of 2,4,6-TCP and PCP were associated with significantly lower birth weight in boys but not girls. Higher prenatal concentrations of PCP were associated with significantly lower birth length in boys but not girls.	Gestational age, pre-pregnancy BMI, maternal age, parity, gestational weight gain, family income, education, occupation, smoking habits, child sex, sex x log 10-(each CP) concentration	Not serious
Abdel Hamid et al. (2020)	Egypt	Cross-sectional	81	Pregnant women for a hospital in Cairo	Not reported	Blood and cord blood samples	Prenatally	p,p’-DDT, p,p’-DDE, p, p’-DDD, α-HCH, β-HCH, γ-HCH, δ-HCH, HCB, heptachlor, heptachlor epoxide, endosulfan, endosulfan I, endosulfan II, endosulfansulphate, chlordane, aldrin, aldrin A, dieldrin, endrin, endrinaldehyde, methoxychlor	Birth weight, birth length	No significant associations between prenatal concentrations of examined pesticides and birth weight or birth length	Not accounted for	Serious
Hanke et al. (2003)	Poland	Cross-sectional	104	Pregnant women living in Zadzim district, Central Poland	Residential (agricultural district)	Self-reported	Prenatally and pre-conceptionally	Phenoxyacetic acid derivatives, synthetic pyrethroids, benzene thiosulphonate derivatives, organo-phosphorus compounds, inorganic derivatives of copper	Birth weight	Prenatal exposure to synthetic pyrethroids was associated with significantly lower birth weight	Pregnancy duration, infant gender, maternal age, pre-pregnancy weight, smoking habit, year of birth, women’s direct field work involvement, farming type	Not serious
Jaacks et al. (2019)	Bangladesh	Prospective cohort	289	Pregnant women from two rural districts	Drinking water	Urine sample	Prenatally at <16 weeks’ gestation	2,4-D, TCPY, MDA, IMPY, 4-F-3-PBA, 3-PBA, trans-DCCA	Low birth weight, preterm birth, small-for-gestational age birth weight, length-for-age Z-score at 1 and 2 y, stunting at 1 and 2 y, weight-for-age Z-score at 1 and 2 y, weight-for-length Z-score at 1 and 2 y	Higher prenatal concentrations of 4-nitrophenol were associated with significantly higher risk of small-for-gestational age and preterm birth. Higher prenatal concentrations of 3-PBA were associated with significantly higher risk of small-for-gestational age. Higher prenatal concentrations of IMPY were associated with significantly higher risk of low birth weight. None of the pesticides assessed were associated with birth weight, LAZ at 1 or 2 years of age, or stunting at 1 or 2 years of age in adjusted models.	Household income, maternal education, dietary intake, and infection	Not serious
Jurewicz et al. (2005)	Poland	Cross-sectional	460	Pregnant women who had been working for a period of at least two years in greenhouses	Occupational (greenhouse workers)	Reported by those responsible for greenhouse chemical protection		17 pesticides classified as reproductive and developmental toxins were applied in the greenhouses (mancozeb, fenbutatin-oxide, triforine, benomyl, thiophanate-methyl, zineb, permethrin, bifenthrin, thiram, oxythiquinox, vinclozolin, dinocap, dimethoate, amitraz, diazinon, cyhexatin, propargite)	Low birth weight, term low birth weight, birth weight, preterm birth,	No significant associations between working in greenhouses and any of the outcomes	Maternal weight, age, smoking habits, education and the place of residence (birth weight model only)	Serious
Liu et al. (2016)	China	Prospective cohort	310	Pregnant women and their children 2 years of age living in an agricultural region producing cotton and rice	Residential (agricultural region), indoor insecticide use, occupational (farming)	Urine sample	Prenatally prior to delivery and postnatally when the child was 2 years of age	DMP, DMTP, DMDTP, DEP, DETP, DEDTP	Birth weight, birth length	No significant associations between prenatal exposure to organophosphates and birth weight or length. No differences between boys and girls.	Maternal age, education, gestational age, pregnancy weight gain, pre-pregnancy BMI, parity, delivery mode, child sex, smoking, maternal and paternal work status, family income, cord blood lead values, sampling season, inhabitation	Not serious
Moreno-Banda et al. (2009)	Mexico	Cross-sectional	328	Women with at least one pregnancy in the 10 years prior to the time of the interview who were floriculturists or partners of floricultural workers	Occupational (floriculture)	Self-reported	Prenatally in each trimester	Most commonly used insecticides were endosulphan, diazinon, metamidophos, omethoate, methyl parathion, carbofuran, methomyl, oxamyl, bifenthrin, permethrin, imidacloprid and abamectin. Most commonly used fungicides were benomyl, carbendazim, methyl thiophanate, mancozeb, triadimephon, captan, chlorotalonyl, iprodione, triforine and metalaxyl.	Low birth weight	Exposure to floricultural work at any time during the pregnancy was not associated with low birth weight	Child sex, adverse reproductive history, PON1 192RR	Not serious
Naksen et al. (2015)	Thailand	Prospective cohort	52	Pregnant women who were farmers and lived in Fang district	Occupational (farm workers)	Urine sample	Prenatally	DMP, DMTP, DMDTP, DEP, DETP, DEDTP	Birth weight, birth length	Among mothers with low PON1 activity, maternal sum DEAP (DEP, DETP, DETPD) and DAP (all six) levels were associated with significantly lower birth weight. No significant associations between maternal DAP levels and birth weight or length were observed in the high maternal PON1 activity group.	Maternal age, pre-pregnancy BMI, weight gain and gestational age	Not serious
Rahimi et al. (2020)	Iran	Cross-sectional	645	Women of reproductive	Occupational (greenhouse workers)	Greenhouse workers with ≥1 year work experience and ≥1 year marriage were compared to housewives	Prenatally	Not reported	Low birth weight, preterm birth	Significantly higher prevalence of LWB and PTB was observed among greenhouse workers than housewives.	Not accounted for	Very serious
Silvia et al. (2020)	Argentina	Cross-sectional	53	Pregnant women	Residential (agricultural region)	Residential location	Prenatally during the third trimester	Organophosphates such as chlorpyrifos and azinphos-methyl and carbamates such as carbofuran and pirimicarb are most frequently used in this region.	Birth weight, birth length, low birth weight, preterm birth, small-for-gestational age	No differences in birth weight, birth length, or small for gestational age between women assessed during the spraying season and women assessed during the non-spraying season. Higher proportion of births were low birth weight or preterm in women assessed during the spraying season than in women assessed during the non-spraying season.	Gestational age and child sex in the models for birth weight and length.	Serious
Steinholt et al. (2020)	Cambodia	Cross-sectional	198	Pregnant women	Dietary; residential (living in rice farming communities, insecticide use at home)	Blood and urine samples	Prenatally	HCB, heptachlor, o,p’-DDE, p,p’-DDE, o,p’-DDD, p,p’-DDD, p,p’-DDT, aldrin, mirex	Birth weight, birth length	Higher prenatal concentrations of o, p’-DDD were associated with significantly higher birth weight and significantly lower birth length. Higher prenatal concentrations of p, p’-DDD were associated with significantly lower birth length. Higher prenatal concentrations of Aldrin were associated with significantly higher birth length. Other pesticides showed no significant patterns.	Gestational age, maternal age, parity, BMI, residence area, education, and occupation	Not serious
Toichuev et al. (2018)	Kyrgyzstan	Cohort	508	Pregnant women	Residential (living in a cotton growing region); dietary	Placenta sample	Prenatally	α-HCH, γ-HCH, β-HCH, and δ-HCH; DDT, DDE and DDD; aldrin, dieldrin, and heptachlor	Low birth weight	Higher prevalence of low birth weight was observed in the group of women with detectable levels of organochlorine pesticides relative to the group of women with undetectable levels of organochlorine pesticides.	Not accounted for	Serious
Van Tung et al. (2016)	Vietnam	Cross-sectional	120	Lactating women	Residential (living in areas where chemical herbicides were stored and spilled and aircrafts that sprayed Agent Orange were washed)	Breast milk and saliva samples	Prenatally and postnatally	Dioxin (defined as only PCDDs/PCDFs, not including PCBs)	Birth weight, low birth weight, weight and height at 8–9 and 12–14 weeks	The prevalence of low birth weight was higher among women exposed to dioxin than unexposed women. Dioxin isomers were significantly negatively correlated with birth weight but not with weight or height at 8–9 or 12–14 weeks	Not accounted for	Serious
Wang et al. (2012a)	China	Cross-sectional	503	Pregnant women	Occupational, personal use at home	Self-reported	Prenatally during pregnancy	Not reported	Birth weight, low birth weight	No significant associations with prenatal pesticide exposure.	Household location, maternal race, education, age, child sex, gestational age, age of menarche	Not serious
Wang et al. (2012b)	China	Cross-sectional	187	Pregnant women	Multiple sources	Self-reported; urinary sample	Prenatally	DMP, DMTP, DEP, DETP, DEDTP	Birth weight, birth length	No significant associations with prenatal pesticide exposure.	Gestational age, maternal height, pregnancy weight gain, family income	Not serious
Xu et al. (2017)	China	Cross-sectional	106	Pregnant women	Residential (living in area with high agricultural pollution)	Cord serum	Prenatally at birth	p,p’-DDE, o,p’-DDD, p, p’-DDD, o,p’-DDT, p, p’-DDT	Birth weight, birth length	Higher prenatal concentrations of p, p’-DDD and p,p’-DDT were associated with higher birth weight in adjusted models. No significant associations with birth length	Unadjusted: maternal BMI, age, education Adjusted: maternal age, education, BMI, infant sex, abortion times, parity, weight gain, drinking water	Not serious
Yang et al. (2020)	China	Cross-sectional	102	Pregnant women	Non-occupational	Blood sample	Prenatally	9 organophosphates (propetamphos, phosalone, diazinon, methacrifos, pyrazophos, mecarbam, parathion-methyl, phthalimide, isazofos), 7 organochlorines (alachlor, α-HCH, β-HCH, γ-HCH, δ-HCH, o,p’-DDT p,p’-DDD), 5 carbamates (fenobucarb, pirimicarb, propham, propoxur, isoprocarb), and 16 others (diclobutrazol, dicamba, DDA, atrazine, tetramethrin, chlorothalonil, ethofumesate, furalaxyl, 2-phenyl-phenol, metalaxyl, chlozolinate, diphenamid, triclosan, flutolanil, dicloran, simazine)	Birth weight	Higher prenatal concentrations of β-HCH were associated with significantly lower birth weight	Pre-pregnancy BMI, maternal education, age, smoking, weight gain, drinking, child sex, gestation length, smoking, family income	Not serious
Yang et al. (2021)	China	Prospective cohort	1039	Pregnant women	Multiple sources	Cord serum	Prenatally at birth	α-HCH, β-HCH, γ-HCH, p,p′-DDT, p,p′-DDD, p, p′-DDE	Body mass index Z-score, overweight at birth, 6, 12, and 24 months of age, growth velocity (difference in weight z-score), birth length	Higher prenatal concentrations of β-HCH were associated with significantly higher BMIZ at 12 and 24 months and significantly higher growth velocity from 0 to 12 months. Higher prenatal concentrations of γ-HCH were associated with significantly higher BMIZ at 6 months and higher growth velocity from 0 to 6 months. Higher prenatal concentrations of p, p′-DDE were associated with significantly lower BMIZ at 6 months. Higher prenatal concentrations of p, p′-DDT were associated with higher BMIZ at 12 months. Higher prenatal concentrations of sum DDT were associated with lower BMIZ at 6 months. Higher prenatal concentrations of total HCH were associated with significantly higher growth velocity from 0 to 12 months. No significant associations with overweight or birth length.	Infant gender, maternal age, education, height, smoking, pre-pregnancy BMI, gestational weight gain, parity, duration of breastfeeding	Not serious
Zhang et al. (2018)	China	Prospective cohort	1100	Pregnant women	Not specified	Urine sample	Prenatally	Carbofuranphenol	Birth weight; birth length	No significant associations with prenatal carbofuranphenol levels. No differences between boys and girls.	Gestational duration, maternal age, pre-pregnancy BMI, gestational weight gain, education, parity, pregnancy smoking family income	Not serious

Abbreviations: 2,4-D, 2,4-dichlorophenoxyacetic acid; 4-F-3-PBA, 3-PBA, 3-phenoxybenzoic acid; BMI, body mass index; BMIZ, body mass index Z-score; DAP, dialkylphosphate; DCCA, 3-(2,2-dichlorovinyl)-2,2-dimethylcyclopropane carboxylic acid; DCP, dichlorophenol; DDD, dichlorodiphenyldichloroethane; DDE, dichlorodiphenyldichloroethylene; DDT, dichlorodiphenyltrichloroethane; DEDTP, diethydithiophosphate DEP, diethylphosphate; DETP, diethylthiophosphate; DMDTP, dimethydithiophosphate; DMP, methylphosphate; DMTP, dimethylthiophosphate; HCB, hexachlorobenzene; HCH, hexachlorocyclohexane; IMPY, 2-isopropyl-4-methyl-6-hydroxypyrimidine; LAZ, length-for-age Z-score; LBW, low birth weight; MDA, malathion dicarboxylic acid; PCB, polychlorinated biphenyl; PCDD, polychlorinated dibenzodioxin; PCDF, polychlorinated dibenzofuran; PCP, pentachlorophenol; PON1, Paraoxonase 1; PTB, pre-term birth; SGA, small-for-gestational age; TCP, trichlorophenol; TCPY, 3,5,6-trichloro-2-pyridinol; WAZ, weight-for-age Z-score; WHZ, weight-for-height Z-score.

**Table 2:** Summary of findings on the associations between prenatal pesticide exposure and the four most frequently reported outcomes (birth weight, birth length, low birth weight, and preterm birth).

	Most frequently reported outcomes in included studies														
	Birth weight				Birth length				Low birth weight				Preterm birth		
	Direction	Effect estimate	Refs		Direction	Effect estimate	Refs		Direction	Effect estimate	Refs		Direction	Effect estimate	Refs
Organochlorines	Positive	0.008 to 0.25 SMD	(Arrebola et al., 2016; Bravo et al., 2019; Steinholt et al., 2020; Xu et al., 2017)		Positive	0.21 to 0.25 SMD	(Bravo et al., 2019; Steinholt et al., 2020)		Positive	–	No studies		Positive	No estimate reported	(Cioroiu et al., 2010)
	Negative	-0.009 SMD; -5.81 to -55.14 g	(Anand & Taneja, 2020; X. Yang et al., 2020)		Negative	-0.25 to -0.32 SMD	(Steinholt et al., 2020)		Negative	–	No studies		Negative	No estimate reported	(Cioroiu et al., 2010)
	Null	-32.9 g to 6.09 g	(Abdel Hamid et al., 2020; Fang et al., 2019; H. Guo et al., 2014; Van Tung et al., 2016)		Null	-0.37 to 0.358 cm	(Abdel Hamid et al., 2020; Anand & Taneja, 2020; Arrebola et al., 2016; Fang et al., 2019; Xu et al., 2017)		Null	8 to 13.1 pp	(Toichuev et al., 2018; Van Tung et al., 2016)		Null	–	No studies
Organophosphates	Positive	–	No studies		Positive	–	No studies		Positive	RR 2.13 6 pp	(Jaacks et al., 2019; Silvia et al., 2020)		Positive	RR 1.44 to RR 3.57 6 pp	(Jaacks et al., 2019; Silvia et al., 2020)
	Negative	-170±60 g	(Jaacks et al., 2019)		Negative	-0.372 cm	(Cecchi et al., 2021)		Negative	–	No studies		Negative	–	No studies
	Null	-116 to 135 g	(Cecchi et al., 2021; Hanke et al., 2003; Jaacks et al., 2019; Jurewicz et al., 2005; Liu et al., 2016; Naksen et al., 2015; Silvia et al., 2020; P. Wang et al., 2012; X. Yang et al., 2020)		Null	-0.47 to 0.12 cm	(Liu et al., 2016; Naksen et al., 2015; Silvia et al., 2020; P. Wang et al., 2012)		Null	OR 0.48 2.63 to 6.29 pp	(Jurewicz et al., 2005; Moreno-Banda et al., 2009)		Null	RR 1.17 to RR 0.95 1.63 to 5.8 pp	(Cecchi et al., 2021; Jaacks et al., 2019; Jurewicz et al., 2005)
Pyrethroids	Positive	–	No studies		Positive	–	No studies		Positive	–	No studies		Positive	–	No studies
	Negative	-96.76 to -233.3 g	(Ding et al., 2015; Hanke et al., 2003)		Negative	–	No studies		Negative	–	No studies		Negative	–	No studies
	Null	29 to 102 g	(Jaacks et al., 2019; Jurewicz et al., 2005)		Null	0.13 to 0.25 cm	(Ding et al., 2015)		Null	RR 0.78 2.63 to 6.29 pp	(Jaacks et al., 2019; Jurewicz et al., 2005)		Null	RR 0.99 1.63 to 5.8 pp	(Jaacks et al., 2019; Jurewicz et al., 2005)
Carbamates	Positive	–	No studies		Positive	–	No studies		Positive	–	No studies		Positive	6 pp	(Silvia et al., 2020)
	Negative	–	No studies		Negative	-0.372 cm	(Cecchi et al., 2021)		Negative	–	No studies		Negative	–	No studies
	Null	-3.594 to 0.23 g	(Cecchi et al., 2021; Hanke et al., 2003; Silvia et al., 2020; X. Yang et al., 2020; Zhang et al., 2018)		Null	-0.155 cm	(Zhang et al., 2018)		Null	No estimate reported	(Cecchi et al., 2021)		Null	No estimate reported	(Cecchi et al., 2021)
Chlorophenols non-specific to organochlorines	Positive	–	No studies		Positive	–	No studies		Positive	–	No studies		Null	–	No studies
	Negative	-30 to -37 g	(J. Guo et al., 2016)		Null	-0.01 to -0.14 cm	(Guo et al., 2016)		Negative	–	No studies		Null	–	No studies
	Null	–	No studies		Null	–	No studies		Null	–	No studies		Null	–	No studies

Abbreviations: OR; odds ratio; pp, percentage points; Refs, references; RR, relative risk; SMD, standardised mean difference.

## Data Availability

No data was used for the research described in the article.
