# Supplementary material for: Pesticide exposure and child growth in low- and middle-income countries: A systematic review
Source: Environ Res. Author manuscript; Available in PMC 2023 May 10. (PMC7614514; doi:10.1016/j.envres.2022.114230)
Supplement: Appendix A. Supplementary data [file EMS175075-supplement-Appendix_A__Supplementary_data.docx]

**Supplemental Table 1** Search strategies as implemented in the 10 electronic databases

| **PubMed**  (“Child”[MH] OR “Child, Preschool”[MH] OR “child*”[tiab] OR “Infant”[MH] OR “infant*”[tiab] OR “paediatric”[tiab] OR “pediatric”[tiab] OR “toddler*”[tiab])  AND  (“Pesticides”[MH] OR “pesticide”[All fields] OR “Organophosphates”[MH] OR “organophosphate”[All fields] OR “organophosphorus”[All fields] OR "Organophosphorus Compounds"[MH] OR "Organophosphate Poisoning"[tiab] OR “aryldialkylphosphatase”[tiab] OR "Cholinesterase Inhibitors"[MH] OR "Cholinesterase Reactivators"[MH] OR "poly(thiophene-3-acetic acid" OR "neurotoxic esterase" OR "parathion hydrolase, Flavobacterium balustinum" OR “8- (4- (4- ((hydroxyimino) methyl) -1-pyridiniumyl) butyl) -3- ((3-hydroxy-2-phenylpropanoyl) oxy) -8-methyl-8-azoniabicyclo- (3.2.1) octane”[All fields] OR “isocarbophos” OR “1- ((4- (aminocarbonyl) pyridinio) trimethylene) -2- ((hydroxyimino) methyl) pyridinium” OR “duodote” OR “anilofos” OR “diethylphosphoryloxime” OR "tributylmethyl phosphonium chloride" OR “diclofluanid” OR "chloroacetol phosphate" OR “crufomate” OR “phosphorylphosphatase” OR "fenitrothion phosphatase" OR "phosphoramidic acid" OR “4- AND (4-nitrobenzyl) pyridine” OR “soman” OR “sarin” OR "Pralidoxime Compounds"[MH] OR “armin” OR “dichlorvos” OR “chlorfenvinphos” OR “armin” OR "Carbamyl Phosphate"[MH] OR “iphos” OR “mevinphos” OR “monocrotophos” OR “naled” OR “azinphosmethyl” OR “chlorpyrifos” OR “coumaphos” OR “cystaphos” OR “diazinon” OR “dimethoate” OR “disulfoton” OR “fenitrothion” OR “fenthion” OR “fonofos” OR “leptophos” OR “malathion” OR “parathion” OR "methyl parathion"[MH] OR "Phenylphosphonothioic Acid, 2-Ethyl 2-(4-Nitrophenyl) Ester"[MH] OR “phorate” OR “phosmet” OR “temefos” OR “paraoxon” OR “phosphamidon” OR “phosphines” OR “trichlorfon” OR “tetrachlorvinphos” OR “tetraisopropylpyrophosphamide” OR “dimethylphosphate” OR “dimethylthiophosphate” OR “dimethyldithiophosphate” OR “diethylphosphate” OR "dimethyl thiophosphate" OR "dimethyl dithiophosphate" OR "dimethyl phosphate" OR "dimethyl thiophosphate" OR "dimethyl dithiophosphate" OR "diethyl phosphate" OR "diethyl thiophosphate" OR "diethyl dithiophosphate" OR "Ethyl chlorpyriphos" OR "methyl chlorpyriphos" OR “PNP” OR “tcpy” OR “3,5,6-trichloro-2-pyridinol”)  AND  (“child growth”[tiab] OR “linear growth”[tiab] OR “stunting”[tiab] OR “length-for-age Z-score”[tiab] OR “height-for-age Z-score”[tiab] OR “HAZ”[tiab] OR “LAZ”[tiab] OR “weight-for-age Z-score”[tiab] OR “WAZ”[tiab] OR “weight-for-length Z-score”[tiab] OR “WLZ”[tiab] OR “weight-for-height Z-score”[tiab] OR “WHZ”[tiab] OR “overweight”[tiab] OR “overweight”[MH] OR “body mass index Z-score”[tiab] OR “BMIZ”[tiab] OR “growth retardation”[tiab] OR “growth faltering”[tiab] OR “Growth Disorders”[MH] OR “Premature Birth”[MH] OR “Infant, premature”[MH] OR “pre?term”[tiab] OR “pre?mature”[tiab] OR “Infant, Low Birth Weight”[MH] OR “low birth?weight”[tiab] OR “LBW”[tiab] OR “Infant, Very Low Birth Weight”[MH] OR “very low birth?weight”[tiab] OR “VLBW” [tiab] OR “Infant, Small for Gestational Age”[MH] OR “small for gestational age”[tiab]) |
| --- |

**Cochrane Library**

(“Child” OR “Child, Preschool” OR “child” OR “Infant” OR “infant” OR “paediatric” OR “pediatric” OR “toddler”)

AND

(“Pesticides” OR “pesticide” OR “Organophosphates” OR “organophosphate” OR “organophosphorus” OR "Organophosphorus Compounds" OR "Organophosphate Poisoning" OR “aryldialkylphosphatase” OR "Cholinesterase Inhibitors" OR "Cholinesterase Reactivators" OR "neurotoxic esterase" OR "parathion hydrolase, Flavobacterium balustinum" OR “isocarbophos” OR “duodote” OR “anilofos” OR “diethylphosphoryloxime” OR "tributylmethyl phosphonium chloride" OR “diclofluanid” OR "chloroacetol phosphate" OR “crufomate” OR “phosphorylphosphatase” OR "fenitrothion phosphatase" OR "phosphoramidic acid" OR “soman” OR “sarin” OR "Pralidoxime Compounds" OR “armin” OR “dichlorvos” OR “chlorfenvinphos” OR “armin” OR "Carbamyl Phosphate" OR “iphos” OR “mevinphos” OR “monocrotophos” OR “naled” OR “azinphosmethyl” OR “chlorpyrifos” OR “coumaphos” OR “cystaphos” OR “diazinon” OR “dimethoate” OR “disulfoton” OR “fenitrothion” OR “fenthion” OR “fonofos” OR “leptophos” OR “malathion” OR “parathion” OR "methyl parathion" OR “phorate” OR “phosmet” OR “temefos” OR “paraoxon” OR “phosphamidon” OR “phosphines” OR “trichlorfon” OR “tetrachlorvinphos” OR “tetraisopropylpyrophosphamide” OR “dimethylphosphate” OR “dimethylthiophosphate” OR “dimethyldithiophosphate” OR “diethylphosphate” OR "dimethyl thiophosphate" OR "dimethyl dithiophosphate" OR "dimethyl phosphate" OR "dimethyl thiophosphate" OR "dimethyl dithiophosphate" OR "diethyl phosphate" OR "diethyl thiophosphate" OR "diethyl dithiophosphate" OR "Ethyl chlorpyriphos" OR "methyl chlorpyriphos" OR “PNP” OR “tcpy”)

AND

(“child growth” OR “linear growth” OR “stunting” OR “length-for-age Z-score” OR “height-for-age Z-score” OR “HAZ” OR “LAZ” OR “weight-for-age Z-score” OR “WAZ” OR “weight-for-length Z-score” OR “WLZ” OR “weight-for-height Z-score” OR “WHZ” OR “overweight” OR “overweight” OR “body mass index Z-score” OR “BMIZ” OR “growth retardation” OR “growth faltering” OR “Growth Disorders” OR “Premature Birth” OR “Infant, premature” OR “preterm” OR “pre-term” OR “premature” OR “pre-mature” OR “Infant, Low Birth Weight” OR “low birthweight” OR “low birth weight” OR “LBW” OR “Infant, Very Low Birth Weight” OR “very low birthweight” OR “very low birth weight” OR “VLBW” OR “Infant, Small for Gestational Age” OR “small for gestational age”)

**Embase**

(Child OR Child, Preschool OR child* OR Infant OR infant* OR paediatric OR pediatric OR toddler*)

AND

(Pesticides OR pesticide OR Organophosphates OR organophosphate OR organophosphorus OR Organophosphorus Compounds OR Organophosphate Poisoning OR aryldialkylphosphatase OR Cholinesterase Inhibitors OR Cholinesterase Reactivators OR neurotoxic esterase OR parathion hydrolase, Flavobacterium balustinum OR isocarbophos OR duodote OR anilofos OR diethylphosphoryloxime OR tributylmethyl phosphonium chloride OR diclofluanid OR chloroacetol phosphate OR crufomate OR phosphorylphosphatase OR fenitrothion phosphatase OR phosphoramidic acid OR soman OR sarin OR Pralidoxime Compounds OR armin OR dichlorvos OR chlorfenvinphos OR armin OR Carbamyl Phosphate OR iphos OR mevinphos OR monocrotophos OR naled OR azinphosmethyl OR chlorpyrifos OR coumaphos OR cystaphos OR diazinon OR dimethoate OR disulfoton OR fenitrothion OR fenthion OR fonofos OR leptophos OR malathion OR parathion OR methyl parathion OR phorate OR phosmet OR temefos OR paraoxon OR phosphamidon OR phosphines OR trichlorfon OR tetrachlorvinphos OR tetraisopropylpyrophosphamide OR dimethylphosphate OR dimethylthiophosphate OR dimethyldithiophosphate OR diethylphosphate OR dimethyl thiophosphate OR dimethyl dithiophosphate OR dimethyl phosphate OR dimethyl thiophosphate OR dimethyl dithiophosphate OR diethyl phosphate OR diethyl thiophosphate OR diethyl dithiophosphate OR Ethyl chlorpyriphos OR methyl chlorpyriphos OR PNP OR tcpy)

AND

(child growth OR linear growth OR stunting OR length-for-age Z-score OR height-for-age Z-score OR HAZ OR LAZ OR weight-for-age Z-score OR WAZ OR weight-for-length Z-score OR WLZ OR weight-for-height Z-score OR WHZ OR overweight OR overweight OR body mass index Z-score OR BMIZ OR growth retardation OR growth faltering OR Growth Disorders OR Premature Birth OR Infant, premature OR preterm OR pre-term OR premature OR pre-mature OR Infant, Low Birth Weight OR low birthweight OR low birth weight OR LBW OR Infant, Very Low Birth Weight OR very low birthweight OR very low birth weight OR VLBW OR Infant, Small for Gestational Age OR small for gestational age)

**Scopus**

(Child OR Child, Preschool OR child* OR Infant OR infant* OR paediatric OR pediatric OR toddler*)

AND

(Pesticides OR pesticide OR Organophosphates OR organophosphate OR organophosphorus OR Organophosphorus Compounds OR Organophosphate Poisoning OR aryldialkylphosphatase OR Cholinesterase Inhibitors OR Cholinesterase Reactivators OR neurotoxic esterase OR parathion hydrolase, Flavobacterium balustinum OR isocarbophos OR duodote OR anilofos OR diethylphosphoryloxime OR tributylmethyl phosphonium chloride OR diclofluanid OR chloroacetol phosphate OR crufomate OR phosphorylphosphatase OR fenitrothion phosphatase OR phosphoramidic acid OR soman OR sarin OR Pralidoxime Compounds OR armin OR dichlorvos OR chlorfenvinphos OR armin OR Carbamyl Phosphate OR iphos OR mevinphos OR monocrotophos OR naled OR azinphosmethyl OR chlorpyrifos OR coumaphos OR cystaphos OR diazinon OR dimethoate OR disulfoton OR fenitrothion OR fenthion OR fonofos OR leptophos OR malathion OR parathion OR methyl parathion OR phorate OR phosmet OR temefos OR paraoxon OR phosphamidon OR phosphines OR trichlorfon OR tetrachlorvinphos OR tetraisopropylpyrophosphamide OR dimethylphosphate OR dimethylthiophosphate OR dimethyldithiophosphate OR diethylphosphate OR dimethyl thiophosphate OR dimethyl dithiophosphate OR dimethyl phosphate OR dimethyl thiophosphate OR dimethyl dithiophosphate OR diethyl phosphate OR diethyl thiophosphate OR diethyl dithiophosphate OR Ethyl chlorpyriphos OR methyl chlorpyriphos OR PNP OR tcpy)

AND

(child growth OR linear growth OR stunting OR length-for-age Z-score OR height-for-age Z-score OR HAZ OR LAZ OR weight-for-age Z-score OR WAZ OR weight-for-length Z-score OR WLZ OR weight-for-height Z-score OR WHZ OR overweight OR overweight OR body mass index Z-score OR BMIZ OR growth retardation OR growth faltering OR Growth Disorders OR Premature Birth OR Infant, premature OR preterm OR pre-term OR premature OR pre-mature OR Infant, Low Birth Weight OR low birthweight OR low birth weight OR LBW OR Infant, Very Low Birth Weight OR very low birthweight OR very low birth weight OR VLBW OR Infant, Small for Gestational Age OR small for gestational age)

**LILACS**

(Child OR Child, Preschool OR child* OR Infant OR infant* OR paediatric OR pediatric OR toddler*)

AND

(Pesticides OR pesticide OR Organophosphates OR organophosphate OR organophosphorus OR Organophosphorus Compounds OR Organophosphate Poisoning OR aryldialkylphosphatase OR Cholinesterase Inhibitors OR Cholinesterase Reactivators OR neurotoxic esterase OR parathion hydrolase, Flavobacterium balustinum OR isocarbophos OR duodote OR anilofos OR diethylphosphoryloxime OR tributylmethyl phosphonium chloride OR diclofluanid OR chloroacetol phosphate OR crufomate OR phosphorylphosphatase OR fenitrothion phosphatase OR phosphoramidic acid OR soman OR sarin OR Pralidoxime Compounds OR armin OR dichlorvos OR chlorfenvinphos OR armin OR Carbamyl Phosphate OR iphos OR mevinphos OR monocrotophos OR naled OR azinphosmethyl OR chlorpyrifos OR coumaphos OR cystaphos OR diazinon OR dimethoate OR disulfoton OR fenitrothion OR fenthion OR fonofos OR leptophos OR malathion OR parathion OR methyl parathion OR phorate OR phosmet OR temefos OR paraoxon OR phosphamidon OR phosphines OR trichlorfon OR tetrachlorvinphos OR tetraisopropylpyrophosphamide OR dimethylphosphate OR dimethylthiophosphate OR dimethyldithiophosphate OR diethylphosphate OR dimethyl thiophosphate OR dimethyl dithiophosphate OR dimethyl phosphate OR dimethyl thiophosphate OR dimethyl dithiophosphate OR diethyl phosphate OR diethyl thiophosphate OR diethyl dithiophosphate OR Ethyl chlorpyriphos OR methyl chlorpyriphos OR PNP OR tcpy)

AND

(child growth OR linear growth OR stunting OR length-for-age Z-score OR height-for-age Z-score OR HAZ OR LAZ OR weight-for-age Z-score OR WAZ OR weight-for-length Z-score OR WLZ OR weight-for-height Z-score OR WHZ OR overweight OR overweight OR body mass index Z-score OR BMIZ OR growth retardation OR growth faltering OR Growth Disorders OR Premature Birth OR Infant, premature OR preterm OR pre-term OR premature OR pre-mature OR Infant, Low Birth Weight OR low birthweight OR low birth weight OR LBW OR Infant, Very Low Birth Weight OR very low birthweight OR very low birth weight OR VLBW OR Infant, Small for Gestational Age OR small for gestational age)

**Web of Science**

(Child OR Child, Preschool OR child* OR Infant OR infant* OR paediatric OR pediatric OR toddler*)

AND

(Pesticides OR pesticide OR Organophosphates OR organophosphate OR organophosphorus OR Organophosphorus Compounds OR Organophosphate Poisoning OR aryldialkylphosphatase OR Cholinesterase Inhibitors OR Cholinesterase Reactivators OR neurotoxic esterase OR parathion hydrolase, Flavobacterium balustinum OR isocarbophos OR duodote OR anilofos OR diethylphosphoryloxime OR tributylmethyl phosphonium chloride OR diclofluanid OR chloroacetol phosphate OR crufomate OR phosphorylphosphatase OR fenitrothion phosphatase OR phosphoramidic acid OR soman OR sarin OR Pralidoxime Compounds OR armin OR dichlorvos OR chlorfenvinphos OR armin OR Carbamyl Phosphate OR iphos OR mevinphos OR monocrotophos OR naled OR azinphosmethyl OR chlorpyrifos OR coumaphos OR cystaphos OR diazinon OR dimethoate OR disulfoton OR fenitrothion OR fenthion OR fonofos OR leptophos OR malathion OR parathion OR methyl parathion OR phorate OR phosmet OR temefos OR paraoxon OR phosphamidon OR phosphines OR trichlorfon OR tetrachlorvinphos OR tetraisopropylpyrophosphamide OR dimethylphosphate OR dimethylthiophosphate OR dimethyldithiophosphate OR diethylphosphate OR dimethyl thiophosphate OR dimethyl dithiophosphate OR dimethyl phosphate OR dimethyl thiophosphate OR dimethyl dithiophosphate OR diethyl phosphate OR diethyl thiophosphate OR diethyl dithiophosphate OR Ethyl chlorpyriphos OR methyl chlorpyriphos OR PNP OR tcpy)

AND

(child growth OR linear growth OR stunting OR length-for-age Z-score OR height-for-age Z-score OR HAZ OR LAZ OR weight-for-age Z-score OR WAZ OR weight-for-length Z-score OR WLZ OR weight-for-height Z-score OR WHZ OR overweight OR overweight OR body mass index Z-score OR BMIZ OR growth retardation OR growth faltering OR Growth Disorders OR Premature Birth OR Infant, premature OR preterm OR pre-term OR premature OR pre-mature OR Infant, Low Birth Weight OR low birthweight OR low birth weight OR LBW OR Infant, Very Low Birth Weight OR very low birthweight OR very low birth weight OR VLBW OR Infant, Small for Gestational Age OR small for gestational age)

**CAB Abstracts**

(Child OR Child, Preschool OR child* OR Infant OR infant* OR paediatric OR pediatric OR toddler*)

AND

(Pesticides OR pesticide OR Organophosphates OR organophosphate OR organophosphorus OR Organophosphorus Compounds OR Organophosphate Poisoning OR aryldialkylphosphatase OR Cholinesterase Inhibitors OR Cholinesterase Reactivators OR neurotoxic esterase OR parathion hydrolase, Flavobacterium balustinum OR isocarbophos OR duodote OR anilofos OR diethylphosphoryloxime OR tributylmethyl phosphonium chloride OR diclofluanid OR chloroacetol phosphate OR crufomate OR phosphorylphosphatase OR fenitrothion phosphatase OR phosphoramidic acid OR soman OR sarin OR Pralidoxime Compounds OR armin OR dichlorvos OR chlorfenvinphos OR armin OR Carbamyl Phosphate OR iphos OR mevinphos OR monocrotophos OR naled OR azinphosmethyl OR chlorpyrifos OR coumaphos OR cystaphos OR diazinon OR dimethoate OR disulfoton OR fenitrothion OR fenthion OR fonofos OR leptophos OR malathion OR parathion OR methyl parathion OR phorate OR phosmet OR temefos OR paraoxon OR phosphamidon OR phosphines OR trichlorfon OR tetrachlorvinphos OR tetraisopropylpyrophosphamide OR dimethylphosphate OR dimethylthiophosphate OR dimethyldithiophosphate OR diethylphosphate OR dimethyl thiophosphate OR dimethyl dithiophosphate OR dimethyl phosphate OR dimethyl thiophosphate OR dimethyl dithiophosphate OR diethyl phosphate OR diethyl thiophosphate OR diethyl dithiophosphate OR Ethyl chlorpyriphos OR methyl chlorpyriphos OR PNP OR tcpy)

AND

(child growth OR linear growth OR stunting OR length-for-age Z-score OR height-for-age Z-score OR HAZ OR LAZ OR weight-for-age Z-score OR WAZ OR weight-for-length Z-score OR WLZ OR weight-for-height Z-score OR WHZ OR overweight OR overweight OR body mass index Z-score OR BMIZ OR growth retardation OR growth faltering OR Growth Disorders OR Premature Birth OR Infant, premature OR preterm OR pre-term OR premature OR pre-mature OR Infant, Low Birth Weight OR low birthweight OR low birth weight OR LBW OR Infant, Very Low Birth Weight OR very low birthweight OR very low birth weight OR VLBW OR Infant, Small for Gestational Age OR small for gestational age)

**Global Health (CABI)**

(Child OR Child, Preschool OR child* OR Infant OR infant* OR paediatric OR pediatric OR toddler*)

AND

(Pesticides OR pesticide OR Organophosphates OR organophosphate OR organophosphorus OR Organophosphorus Compounds OR Organophosphate Poisoning OR aryldialkylphosphatase OR Cholinesterase Inhibitors OR Cholinesterase Reactivators OR neurotoxic esterase OR parathion hydrolase, Flavobacterium balustinum OR isocarbophos OR duodote OR anilofos OR diethylphosphoryloxime OR tributylmethyl phosphonium chloride OR diclofluanid OR chloroacetol phosphate OR crufomate OR phosphorylphosphatase OR fenitrothion phosphatase OR phosphoramidic acid OR soman OR sarin OR Pralidoxime Compounds OR armin OR dichlorvos OR chlorfenvinphos OR armin OR Carbamyl Phosphate OR iphos OR mevinphos OR monocrotophos OR naled OR azinphosmethyl OR chlorpyrifos OR coumaphos OR cystaphos OR diazinon OR dimethoate OR disulfoton OR fenitrothion OR fenthion OR fonofos OR leptophos OR malathion OR parathion OR methyl parathion OR phorate OR phosmet OR temefos OR paraoxon OR phosphamidon OR phosphines OR trichlorfon OR tetrachlorvinphos OR tetraisopropylpyrophosphamide OR dimethylphosphate OR dimethylthiophosphate OR dimethyldithiophosphate OR diethylphosphate OR dimethyl thiophosphate OR dimethyl dithiophosphate OR dimethyl phosphate OR dimethyl thiophosphate OR dimethyl dithiophosphate OR diethyl phosphate OR diethyl thiophosphate OR diethyl dithiophosphate OR Ethyl chlorpyriphos OR methyl chlorpyriphos OR PNP OR tcpy)

AND

(child growth OR linear growth OR stunting OR length-for-age Z-score OR height-for-age Z-score OR HAZ OR LAZ OR weight-for-age Z-score OR WAZ OR weight-for-length Z-score OR WLZ OR weight-for-height Z-score OR WHZ OR overweight OR overweight OR body mass index Z-score OR BMIZ OR growth retardation OR growth faltering OR Growth Disorders OR Premature Birth OR Infant, premature OR preterm OR pre-term OR premature OR pre-mature OR Infant, Low Birth Weight OR low birthweight OR low birth weight OR LBW OR Infant, Very Low Birth Weight OR very low birthweight OR very low birth weight OR VLBW OR Infant, Small for Gestational Age OR small for gestational age)

**Global Index Medicus**

(Child OR Child, Preschool OR child* OR Infant OR infant* OR paediatric OR pediatric OR toddler*)

AND

(Pesticides OR pesticide OR Organophosphates OR organophosphate OR organophosphorus OR Organophosphorus Compounds OR Organophosphate Poisoning OR aryldialkylphosphatase OR Cholinesterase Inhibitors OR Cholinesterase Reactivators OR neurotoxic esterase OR parathion hydrolase, Flavobacterium balustinum OR isocarbophos OR duodote OR anilofos OR diethylphosphoryloxime OR tributylmethyl phosphonium chloride OR diclofluanid OR chloroacetol phosphate OR crufomate OR phosphorylphosphatase OR fenitrothion phosphatase OR phosphoramidic acid OR soman OR sarin OR Pralidoxime Compounds OR armin OR dichlorvos OR chlorfenvinphos OR armin OR Carbamyl Phosphate OR iphos OR mevinphos OR monocrotophos OR naled OR azinphosmethyl OR chlorpyrifos OR coumaphos OR cystaphos OR diazinon OR dimethoate OR disulfoton OR fenitrothion OR fenthion OR fonofos OR leptophos OR malathion OR parathion OR methyl parathion OR phorate OR phosmet OR temefos OR paraoxon OR phosphamidon OR phosphines OR trichlorfon OR tetrachlorvinphos OR tetraisopropylpyrophosphamide OR dimethylphosphate OR dimethylthiophosphate OR dimethyldithiophosphate OR diethylphosphate OR dimethyl thiophosphate OR dimethyl dithiophosphate OR dimethyl phosphate OR dimethyl thiophosphate OR dimethyl dithiophosphate OR diethyl phosphate OR diethyl thiophosphate OR diethyl dithiophosphate OR Ethyl chlorpyriphos OR methyl chlorpyriphos OR PNP OR tcpy)

AND

(child growth OR linear growth OR stunting OR length-for-age Z-score OR height-for-age Z-score OR HAZ OR LAZ OR weight-for-age Z-score OR WAZ OR weight-for-length Z-score OR WLZ OR weight-for-height Z-score OR WHZ OR overweight OR overweight OR body mass index Z-score OR BMIZ OR growth retardation OR growth faltering OR Growth Disorders OR Premature Birth OR Infant, premature OR preterm OR pre-term OR premature OR pre-mature OR Infant, Low Birth Weight OR low birthweight OR low birth weight OR LBW OR Infant, Very Low Birth Weight OR very low birthweight OR very low birth weight OR VLBW OR Infant, Small for Gestational Age OR small for gestational age)

**SciELO**

(Child OR Child, Preschool OR child* OR Infant OR infant* OR paediatric OR pediatric OR toddler*)

AND

(Pesticides OR pesticide OR Organophosphates OR organophosphate OR organophosphorus OR Organophosphorus Compounds OR Organophosphate Poisoning OR aryldialkylphosphatase OR Cholinesterase Inhibitors OR Cholinesterase Reactivators OR neurotoxic esterase OR parathion hydrolase, Flavobacterium balustinum OR isocarbophos OR duodote OR anilofos OR diethylphosphoryloxime OR tributylmethyl phosphonium chloride OR diclofluanid OR chloroacetol phosphate OR crufomate OR phosphorylphosphatase OR fenitrothion phosphatase OR phosphoramidic acid OR soman OR sarin OR Pralidoxime Compounds OR armin OR dichlorvos OR chlorfenvinphos OR armin OR Carbamyl Phosphate OR iphos OR mevinphos OR monocrotophos OR naled OR azinphosmethyl OR chlorpyrifos OR coumaphos OR cystaphos OR diazinon OR dimethoate OR disulfoton OR fenitrothion OR fenthion OR fonofos OR leptophos OR malathion OR parathion OR methyl parathion OR phorate OR phosmet OR temefos OR paraoxon OR phosphamidon OR phosphines OR trichlorfon OR tetrachlorvinphos OR tetraisopropylpyrophosphamide OR dimethylphosphate OR dimethylthiophosphate OR dimethyldithiophosphate OR diethylphosphate OR dimethyl thiophosphate OR dimethyl dithiophosphate OR dimethyl phosphate OR dimethyl thiophosphate OR dimethyl dithiophosphate OR diethyl phosphate OR diethyl thiophosphate OR diethyl dithiophosphate OR Ethyl chlorpyriphos OR methyl chlorpyriphos OR PNP OR tcpy)

AND

(child growth OR linear growth OR stunting OR length-for-age Z-score OR height-for-age Z-score OR HAZ OR LAZ OR weight-for-age Z-score OR WAZ OR weight-for-length Z-score OR WLZ OR weight-for-height Z-score OR WHZ OR overweight OR overweight OR body mass index Z-score OR BMIZ OR growth retardation OR growth faltering OR Growth Disorders OR Premature Birth OR Infant, premature OR preterm OR pre-term OR premature OR pre-mature OR Infant, Low Birth Weight OR low birthweight OR low birth weight OR LBW OR Infant, Very Low Birth Weight OR very low birthweight OR very low birth weight OR VLBW OR Infant, Small for Gestational Age OR small for gestational age)

**Supplemental Table 2** GRADE Summary of Findings Table

| **Certainty assessment** | | | | | | | | | | | | | | | | | |
| --- | --- | --- | --- | --- | --- | --- | --- | --- | --- | --- | --- | --- | --- | --- | --- | --- | --- |
| **Participants (studies) Follow-up** | **Risk of bias** | | | **Inconsistency** | | | **Indirectness** | | | **Imprecision** | | | **Publication bias** | | **Overall certainty of evidence** | | |
|  |  |  |  |  |  |  |  |  |  |  |  |  |  |  |  |  |  |
| **Birth weight** | | |  | | |  | | |  | | |  | |  | | |  |
| 8062 (24 observational studies) | not serious | | | very serious^a^ | | | serious^b^ | | | very serious^d^ | | | all plausible residual confounding would suggest spurious effect, while no effect was observed | | ⨁◯◯◯ Very low | | |
| **Birth length** | |  | | |  | | |  | | |  | | |  | |  |  |
| 5985 (15 observational studies) | not serious | | | very serious^a^ | | | serious^b^ | | | very serious^d^ | | | all plausible residual confounding would suggest spurious effect, while no effect was observed | | ⨁◯◯◯ Very low | | |
| **Low birth weight (<2500 g)** | | | | | | | | | | | | | | | |  |  |
| 3682 (9 observational studies) | serious | | | very serious^a^ | | | very serious^c^ | | | very serious^d^ | | | all plausible residual confounding would suggest spurious effect, while no effect was observed | | ⨁◯◯◯ Very low | | |
| **Preterm birth (<37 weeks' gestation)** | | | | | | | | | | | | | | | |  |  |
| 2286 (6 observational studies) | very serious | | | very serious^a^ | | | very serious^c^ | | | very serious^d^ | | | all plausible residual confounding would suggest spurious effect, while no effect was observed | | ⨁◯◯◯ Very low | | |

1. Substantial clinical and methodological heterogeneity. Studies that report measures of uncertainty also indicate substantial inconsistency of effect estimates.
2. Studies limited to particular participants and settings.
3. Studies limited to particular participants and settings; comparators were not highly applicable, e.g., not directly exposed.
4. Sample sizes vary widely with most studies having small sample sizes <300 participants. Measures of uncertainty not reported in all studies. Those that report measures of uncertainty indicate high imprecision (e.g., wide CIs, large SEs).
